# Inability to sustain intraphagolysosomal killing of *Staphylococcus aureus* predisposes to bacterial persistence in macrophages

**DOI:** 10.1111/cmi.12485

**Published:** 2015-09-02

**Authors:** Jamil Jubrail, Paul Morris, Martin A. Bewley, Simon Stoneham, Simon A. Johnston, Simon J. Foster, Andrew A. Peden, Robert C. Read, Helen M. Marriott, David H. Dockrell

**Affiliations:** ^1^ Department of Infection and ImmunityUniversity of SheffieldSheffieldUK; ^2^ The Florey InstituteUniversity of SheffieldSheffieldUK; ^3^ Department of Molecular Biology and BiotechnologyUniversity of SheffieldSheffieldUK; ^4^ Department of Biomedical SciencesUniversity of SheffieldSheffieldUK; ^5^ Academic Unit of Clinical and Experimental SciencesUniversity of Southampton Medical SchoolSouthamptonUK; ^6^ Academic Directorate of Communicable DiseasesSheffield Teaching HospitalsSheffieldUK

**Keywords:** microbial‐cell interaction, Staphylococci, antimicrobial, immunology

## Abstract

Macrophages are critical effectors of the early innate response to bacteria in tissues. Phagocytosis and killing of bacteria are interrelated functions essential for bacterial clearance but the rate‐limiting step when macrophages are challenged with large numbers of the major medical pathogen *Staphylococcus aureus* is unknown. We show that macrophages have a finite capacity for intracellular killing and fail to match sustained phagocytosis with sustained microbial killing when exposed to large inocula of *S. aureus* (Newman, SH1000 and USA300 strains). *S. aureus* ingestion by macrophages is associated with a rapid decline in bacterial viability immediately after phagocytosis. However, not all bacteria are killed in the phagolysosome, and we demonstrate reduced acidification of the phagolysosome, associated with failure of phagolysosomal maturation and reduced activation of cathepsin D. This results in accumulation of viable intracellular bacteria in macrophages. We show macrophages fail to engage apoptosis‐associated bacterial killing. Ultittop mately macrophages with viable bacteria undergo cell lysis, and viable bacteria are released and can be internalized by other macrophages. We show that cycles of lysis and reuptake maintain a pool of viable intracellular bacteria over time when killing is overwhelmed and demonstrate intracellular persistence in alveolar macrophages in the lungs in a murine model.

## Introduction

*Staphylococcus aureus* is a major cause of infectious disease contributing both to community‐associated and hospital‐associated infection (Fluit *et al*., 2000; Wisplinghoff *et al*., 2004). *S. aureus* is a frequent human colonizer, and the burden of disease is enhanced by the capacity of *S. aureus* to cause bacteraemia, which leads to metastatic infection, with abscesses formed at sites remote to the initial infection (Fowler *et al*., 2003). Accordingly, *S. aureus* bacteraemia is associated with substantial mortality. The emergence of high‐level antimicrobial resistance and in particular methicillin‐resistant *S. aureus* strains further challenges the clinical approach. These infections involve all ages, and the emergence of community‐acquired methicillin‐resistant *S. aureus* has represented a particular medical challenge (Herold *et al*., 1998). Despite the clinical importance of *S. aureus* infection, optimal treatment strategies for common *S. aureus* syndromes, such as bacteraemia, are still debated (Thwaites *et al*., 2011). A more complete understanding of how this pathogen avoids host immune responses is clearly warranted if more effective treatment approaches are to be developed to combat the evolution of antimicrobial resistant strains.

Resident macrophages are the dominant mechanism of bacterial clearance in tissues such as the lungs, while populations such as marginal zone macrophages in the spleen also play an important role in removing particles from the blood (Green and Kass, 1964; Rehm *et al*., 1980; Birjandi *et al*., 2011). *S. aureus* are rapidly phagocytosed by macrophages, and ingestion does not require extensive opsonization, unlike bacteria containing an extensive polysaccharide capsule, instead it relies on non‐opsonic receptors such as the scavenger receptors MARCO and CD36 (Goldstein *et al*., 1974; Jonsson *et al*., 1985; Palecanda *et al*., 1999; Stuart *et al*., 2005). Tissue macrophages are sufficient to clear bacteria from sites such as the lungs because alveolar macrophages can clear bacteria efficiently in mice rendered neutropenic (Rehm *et al*., 1980) and competence in a key microbicidal mechanism for *S. aureus* killing, nicotinamide adenine dinucleotide phosphate‐oxidase (NADPH) oxidase (NOX2), is sufficient to protect against infection when expression is restricted to mononuclear phagocytes (Pizzolla *et al*., 2012). Despite these observations, *S. aureus* express multiple genes, including catalase and superoxide dismutase, that help protect against killing by reactive oxygen species (ROS) inside macrophages (Das and Bishayi, 2010). Macrophages must therefore activate a combination of microbicidal strategies involving ROS, nitric oxide and proteases such as matrix metalloprotease 12 (MMP‐12) to effectively kill ingested bacteria (Shay *et al*., 2003; Houghton *et al*., 2009; Pizzolla *et al*., 2012).

Considerable uncertainty remains about the efficiency of microbicidal mechanisms against *S. aureus* in macrophages and also concerning the ultimate fate of macrophages that have ingested *S. aureus*. Some reports suggest that *S. aureus* induces macrophage apoptosis (Kubota, 2010; Wang *et al*., 2010), while others have described intracellular persistence of *S. aureus* in macrophages rendered resistant to apoptosis by microbial factors (Kubica *et al*., 2008; Koziel *et al*., 2013). We demonstrate that although intracellular microbicidal mechanisms initially kill *S. aureus* rapidly after ingestion, they become progressively exhausted despite ongoing phagocytosis. Phagolysosomal maturation and acidification are incomplete, and macrophages are therefore left with a population of intracellular bacteria, which can persist for prolonged periods within macrophages, escaping other potential mechanisms of bacterial clearance.

## Results

### Macrophages have a finite capacity for intracellular killing of Staphylococcus aureus Newman

To examine how efficiently macrophages kill *S. aureus*, we exposed macrophages to a range of multiplicities of infection (MOI) and measured the capacity of macrophages to phagocytose and kill bacteria. These experiments were performed with THP‐1 cells differentiated using a protocol we have previously shown to produces a cell type that replicates key characteristics of differentiated tissue macrophages which can be appropriately activated in the presence of bacteria to a classically activated phenotype associated with host defence against extracellular bacteria (Daigneault *et al*., 2010). In this model, macrophages were able to contain extracellular growth of bacteria for at least 9 h at an MOI of 0.05, for 4 h at an MOI of 0.5 but were unable to control extracellular replication at an MOI of 5. As shown in Table 1, macrophages internalized bacteria at all doses, and there was a modest increase in total numbers of intracellular bacteria as the MOI increased, even at the highest MOI, when they could not contain extracellular replication. However, the number of non‐viable bacteria appeared to plateau at an MOI of 5, resulting in accumulation of viable bacteria. At lower doses, less than 1% of internalized bacteria were viable, but by an MOI of 5, the figure increased to approximately 20%. Accumulation of viable bacteria at higher MOI was also demonstrated by DRAQ7 staining of intracellular bacteria (Figure S1).

**Table 1:** Macrophages have a finite capacity for intracellular bacterial killing, which is overwhelmed by increasing numbers of bacteria.

MOI	Intracellular bacteria/cell	Total intracellular bacteria	Viable intracellular bacteria	Estimated non‐viable bacteria	Percentage killing
0.05	1.00 ± 0.13	200 000 ± 25 000	1200 ± 1140	199 000 ± 25 500	99.5 ± 0.61
0.50	1.34 ± 0.18	268 000 ± 36 000	2570 ± 1260	266 000 ± 36 900	99.3 ± 0.71
1.00	1.90 ± 0.42	380 000 ± 84 000	2700 ± 1200	377 000 ± 74 200	99.2 ± 0.41
2.00	2.14 ± 0.30	428 000 ± 60 900	3700 ± 1650	422 000 ± 61 500	99.1 ± 0.46
5.00	2.58 ± 1.26	516 000 ± 252 000	105 000 ± 6220	410 000 ± 324 000	79.7 ± 9.38

The number of intracellular bacteria per macrophage, estimated by fluorescence microscopy, multiplied by the total number of macrophages, was used to calculate the total intracellular burden, after 5 h of challenge. The number of viable intracellular bacteria, estimated by surface viable count, was subtracted from this to give an estimate of the non‐viable intracellular bacteria. This was then used to give an estimate of the percentage of intracellular bacteria that were killed. All values are mean ± SD.

### Macrophages capacity for early intracellular killing of Staphylococcus aureus Newman is lost over time

**Figure 1:**
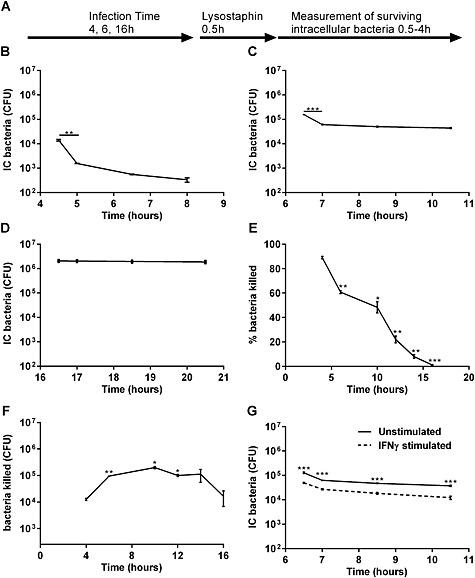
Macrophages challenged with *S. aureus* Newman demonstrate exhaustion of intracellular killing following sustained challenge with bacteria. 
A.Protocol for challenge of differentiated THP‐1 macrophages. Macrophages were challenged for(B) 4, (C) 6 and (D) 16 h, and the kinetics of intracellular (IC) killing estimated, three individual experiments. **p < 0.01;***p < 0.001, Repeated measures analysis of variance (ANOVA) with Sidak's multiple comparisons post‐test comparing the first two time points.(E)Percentage of and (F) absolute numbers of bacteria removed by intracellular killing in the first 1 h following elimination of extracellular bacteria and termination of phagocytosis, three individual experiments. *p < 0.05, ***p < 0.001, One‐way ANOVA with Dunnett's post‐test versus 4 h.G.Differentiated THP‐1 macrophages were cultured with or without IFN‐γ stimulation before challenge with *S. aureus* for 6 h, five individual experiments. ***p < 0.001, two‐way ANOVA with Sidak's multiple comparisons post‐test comparing control with stimulated at each time point.

These results suggest that the ‘bottle‐neck’ in macrophages' capacity to clear extracellular bacteria lies at the level of intracellular bacterial killing. Little is known concerning the kinetics of intracellular killing of *S. aureus* in differentiated macrophages. To measure this, we ‘pulsed’ macrophages with viable bacteria for varying time periods, then killed extracellular bacteria with a lysostaphin ‘chase’ (Tuchscherr *et al*., 2010), allowing us to measure the rate of decline in viable intracellular bacterial numbers (Fig. 1A). As shown in Fig. 1B and consistent with prior data on the kinetics of activation of the NADPH oxidase system in phagocytes (DeLeo *et al*., 1999), the majority of killing occurred in the initial period after ingestion. After this, there was a second more delayed phase of killing during which bacterial viability declined gradually. With increasing durations of exposure to extracellular bacteria, the capacity of macrophages to carry out early intracellular killing declined (Fig. 1B–D). When we looked at the percentage of bacteria killed in the first hour after ingestion, this progressively declined until 16 h after exposure to bacteria, when it became negligible (Fig. 1E). When we looked at the absolute level of bacteria killed, the level increased over the first 6 h but then remained fairly constant at approximately 5 log of bacteria for up to 14 h of exposure to extracellular bacteria after which the absolute number of bacteria killed declined (Fig. 1F). The net effect of incomplete killing is accumulation of viable intracellular bacteria.

Microbicidal killing involves NADPH oxidase stimulation and activation of proteases (Flannagan *et al*., 2009), as well as nitric oxide (Sasaki *et al*., 1998), and these macrophage microbicidal mechanisms are enhanced by IFN‐γ (Cassatella *et al*., 1985; Totemeyer *et al*., 2006). We addressed whether the killing capacity of macrophages could be enhanced by IFN‐γ and whether this would prevent intracellular bacterial persistence. As shown in Fig. 1G, IFN‐γ only modestly increased intracellular killing and did not prevent intracellular persistence, suggesting that failure to increase killing was not due to incomplete priming by IFN‐γ. The failure to clear all intracellular bacteria was not just a function of exposure to large numbers of bacteria because even at an MOI of 0.05 a small number of viable intracellular bacteria were retained and killing became negligible after 16 h of bacterial exposure (Figure S2A‐B). We were also able to confirm that findings were not unique to the Newman strain or THP‐1 macrophages because a phase of initial killing followed by bacterial persistence was also confirmed for SH1000 (Figure S2C–E), a USA300 strain JE2 (Figure S2F–H) and also primary human macrophages (Figure S2I–K).

### The steady initial increase in numbers of intracellular Staphylococcus aureus Newman is the result of sustained macrophage phagocytosis

**Figure 2:**
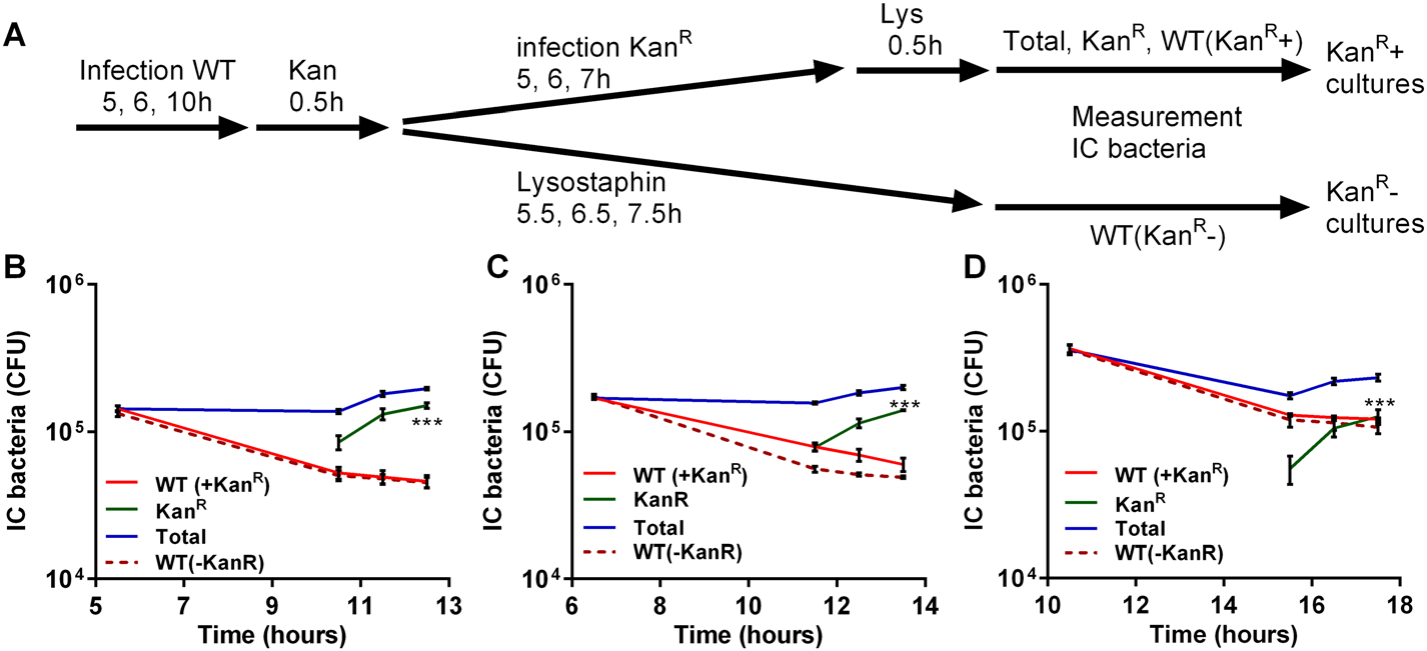
Macrophages continue to internalize *S. aureus* Newman despite failing to maintain intracellular killing. A. Experimental protocol of infection of differentiated THP‐1 macrophages infected with wild type *S. aureus* Newman with (Kan^R^+ cultures) or without (Kan^R^− cultures) an additional pulse of kanamycin‐resistant *S. aureus* (Kan^R^). Initial challenge of (B) 5, (C) 6 or (D) 10 h. WT (in the Kan^R^+ cultures) = CFU Total − CFU Kan^R^, three individual experiments. ***p < 0.001, comparing first and last Kan^R^ point, two‐way analysis of variance with with Dunnett's multiple comparisons test

Having established that the intracellular killing capacity failed to match ongoing phagocytosis, we next investigated whether phagocytosis was sustained after intracellular killing was diminished. The experiments were performed using a variation of the ‘pulse chase’ design where macrophages received a second pulse with a kanamycin‐resistant bacterial strain (Fig. 2A). As shown in Fig. 2B–D, macrophages were able to ingest the second resistant strain at each time point, even at the 16 h time point when intracellular killing was diminished, illustrating that phagocytosis was uncoupled from intracellular killing. Similar results were produced with monocyte‐derived macrophages (MDM) (data not shown).

To confirm that the steady increase in intracellular numbers of viable bacteria was the result of ongoing phagocytosis, not intracellular replication, we performed further experiments using an F‐actin polymerization inhibitor, cytochalasin D, to block the ongoing phagocytosis of *S. aureus* after an initial period of phagocytosis (Brown and Spudich, 1979; DeLoid *et al*., 2009). We performed experiments which illustrated that even at very high MOI bacteria continued to accumulate intracellularly in the absence of cytochalasin D (Figure S3A). Cytochalasin D significantly blocked the accumulation of intracellular bacteria, and over time intracellular numbers declined consistently with intracellular killing (Figure S3B). ROS are thought to contribute to intracellular killing directly or indirectly (Flannagan *et al*., 2009), and when we added a non‐specific antioxidant Trolox [which will inhibit ROS and potentially other species including nitric oxide (Regoli and Winston, 1999)] in the presence of cytochalasin D, we reversed the decline in intracellular numbers but did not see any increase in numbers, further arguing against ongoing intracellular replication. In keeping with the observation that intracellular killing capacity was stressed in this model (as reflected by only a 0.5 log decline in intracellular bacteria when phagocytosis was blocked), we found the addition of Trolox alone had only a minimal effect on the accumulation of viable intracellular bacteria. Collectively, these results suggest that the intracellular accumulation of viable bacteria over this initial period is the result of continued phagocytosis, even when intracellular killing mechanisms are becoming exhausted.

### Staphylococcus aureus Newman containing phagolysosomes demonstrate impaired acidification

Intracellular killing of bacteria in phagolysosomes involves both the generation of antimicrobicidal molecules and appropriate phagolysosomal maturation and acidification (Flannagan *et al*., 2009). The NADPH oxidase system is a key effector of *S. aureus* killing even if the specific role of ROS in microbial killing is a matter of debate (Jackson *et al*., 1995; Reeves *et al*., 2002). Differentiated macrophages, involved in host defence, lack myeloperoxidase gene transcription, limiting production of the more potent ROS (Tobler *et al*., 1988). However, they still generate superoxide, which may contribute to ROS‐dependent *S. aureus* killing in neutrophils or which at least represents a marker of NADPH oxidase activity and an indirect marker of other NADPH oxidase‐regulated killing mechanisms (Hampton *et al*., 1996). As early phagocytosis‐associated killing is linked to NADPH oxidase activation (DeLeo *et al*., 1999), we measured oxidant production with 2′7′‐dichlorofluorescin diacetate (DCF‐DA) and found that there was a dose‐dependent increase in oxidant production following exposure to *S. aureus* at early time points (Figure S4A–B). There was no difference in oxidant production between low dose and high dose *S. aureus* at 16 h (Figure S4C–D), and at both time points, oxidant production was similar to that produced in response to *Streptococcus pneumoniae*, another Gram‐positive bacterium, which is also subjected to intracellular killing in phagocytes (Aberdein *et al*., 2013). Thus, failure to effectively kill higher inocula at the early time point or to maintain killing over time was not the result of reduced oxidant generation or NADPH oxidase activation.

**Figure 3:**
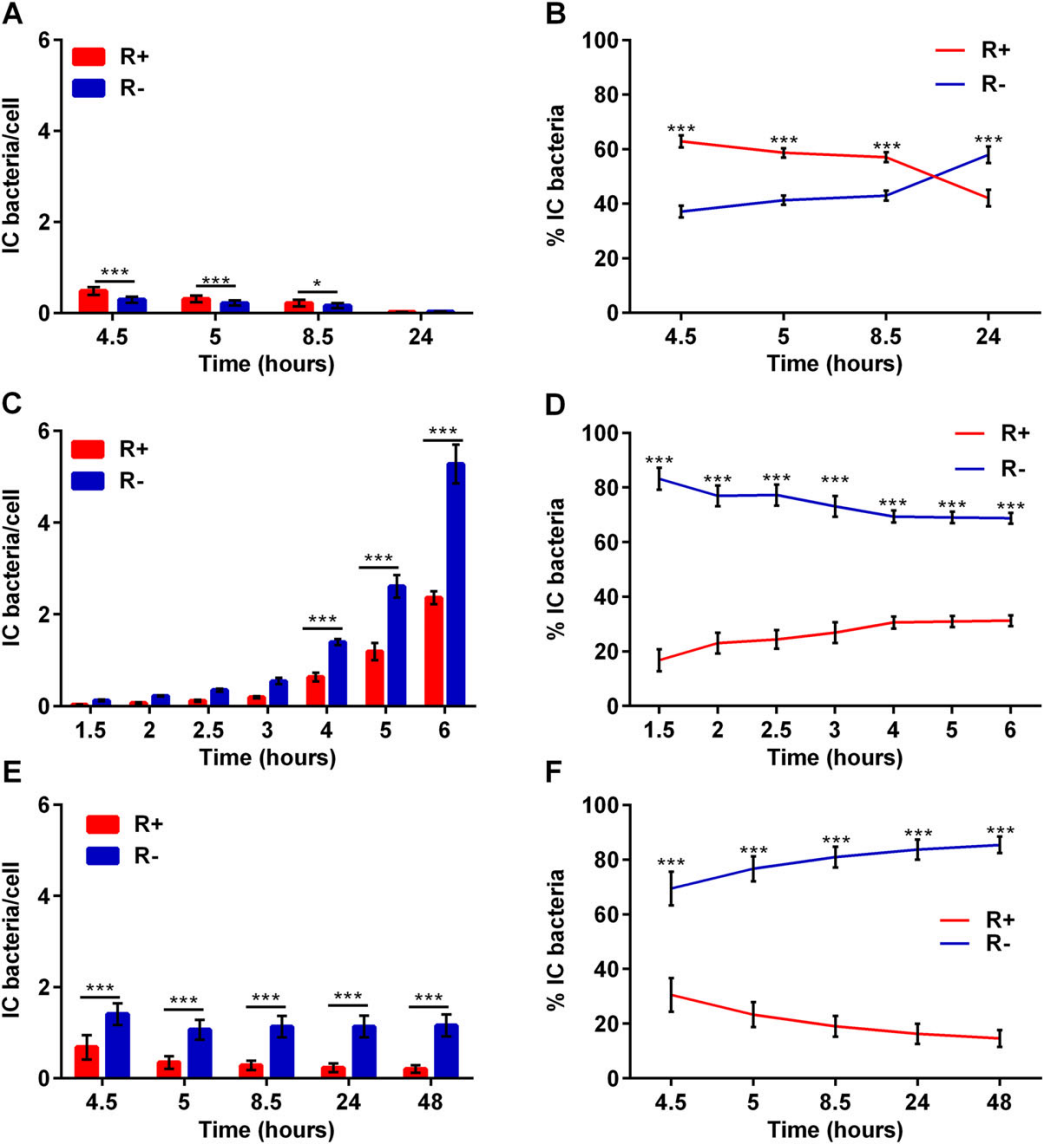
Macrophages traffic *S. aureus* Newman to phagolysosomes that are not appropriately acidified. A, B. Differentiated THP‐1 macrophages were challenged with pHrodo‐labelled *S. pneumoniae* MOI = 5 for 4 h. Cultures were then treated with gentamicin and maintained for up to 24 h post‐infection. A. Number of intracellular (IC) fluorescent (R+) or non‐fluorescent (R−) *S. pneumoniae* per cell. (B) Percentage of intracellular R+/R− *S. pneumoniae*. C–F. Differentiated THP‐1 macrophages were challenged with pHrodo‐labelled *S. aureus* Newman strain MOI = 5 for 1.5–6 h (C–D) or for 4 h followed by treatment with lysostaphin up to 48 h (E–F) and the number of intracellular (C. and E.) or the percentage D and F of R+/R− bacteria were estimated, three individual experiments performed in duplicate. ***p < .001, two‐way ANOVA with Sidak's multiple comparisons post‐test comparing R+ versus R− bacteria at each time point.

Phagolysosomal killing and degradation of bacteria in phagocytes also involves the progressive acidification of the phagosome, which is the result of activation of several pumps that actively import H^+^ ions into the phagolysosome as well as fusion with endosomes that contain high concentrations of H^+^ ions (Flannagan *et al*., 2009). The net effect of these changes is to maintain a low phagolysosome pH despite NADPH oxidase activation, which buffers the acidification of the phagolysosome (Segal *et al*., 1981; Hampton *et al*., 1998). Although most studies on the inter‐relationship between NADPH oxidase and phagolysosomal pH are based on polymorphonuclear leukocytes and monocytes, phagolysosomal acidification is an important feature of macrophages, which aids the killing and digestion of intracellular bacteria (Wolf *et al*., 2011). We next compared the acidification of phagolysosomes containing *S. aureus* using pHrodo‐labelled bacteria (Zarember *et al*., 2012). Staining with pHrodo resulted in fluorescence of bacteria once the pH was ≤6 (Figure S5). Although the majority of *S. pneumoniae* and *Escherichia coli* localized to a phagolysosome whose pH was ≤6 (Fig. 3A–B and Figure S6A–B), a minority of intracellular *S. aureus* localized to an acidified phagosome at early time points, despite increasing levels of internalization (Fig. 3C–D). More prolonged incubation failed to demonstrate increased localization to an acidified endosome (Figs 3E–F and S6C–D). Because initial ROS generation was similar for *S. aureus* and *S. pneumoniae* (Figure S4), differential acidification was not a result of differences in buffering. Furthermore, when we performed ‘pulse chase’ experiments, we found that the reduced acidification of *S. aureus*‐containing phagosomes was sustained over time (Figs 3E–F and S6C–D). To exclude the possibility that higher numbers of ingested *S. aureus* might contribute to this result, we confirmed that only a minority of bacteria were in a low pH compartment even at a low MOI of 0.05 (Figure S6E–F), when numbers of ingested bacteria were more comparable with those for *S. pneumoniae*. Impaired phagolysosomal acidification was also noted with a USA300 strain at both early and later time points with a lysostaphin ‘pulse chase’ (Figure S7). These experiments also suggested that impaired phagolysosomal acidification required live bacteria because heat‐killed bacteria were more likely to translocate to a phagolysosome at lower pH (Figure S7G–H). When we tested the co‐localization of bacteria with Lysotracker, which also fluoresces at an acidic pH, we also found that only approximately 30% of *S. aureus* co‐localized with the low pH phagolysosomes (data not shown). Collectively, these results suggest that *S. aureus* was contained within an endosome that failed to acidify to the same extent as those for other bacteria and that differential buffering effects in association with NADPH oxidase activation did not appear to explain these differences.

### Staphylococcus aureus Newman containing phagosomes fail to mature appropriately

**Figure 4:**
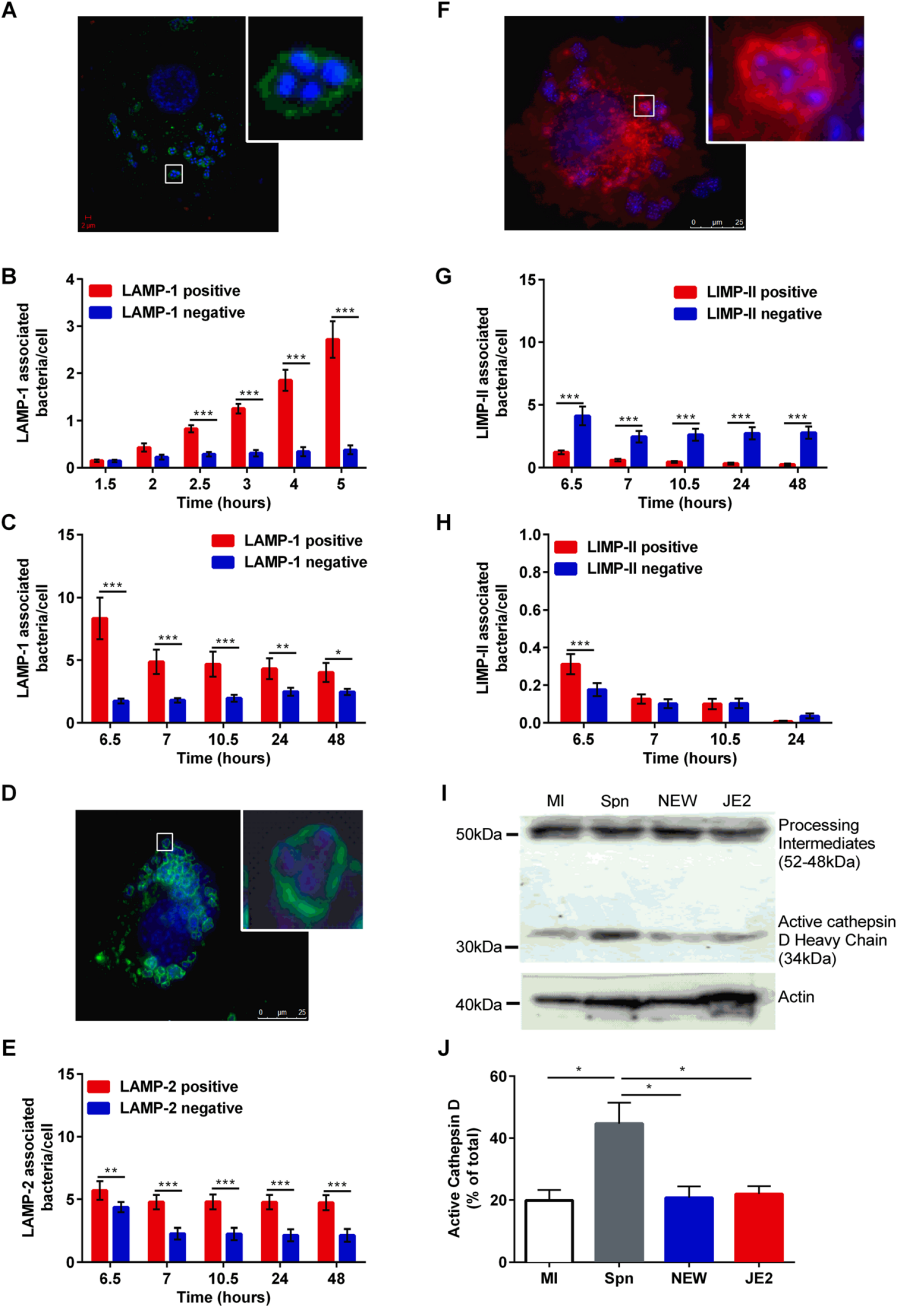
*Staphylococcus aureus* Newman traffic to endosomes that demonstrate incomplete maturation. Differentiated THP‐1 macrophages were challenged with *S. aureus*, MOI = 5. A**.** Representative LAMP‐1 staining by confocal microscopy with enlarged image of macrophage in insert. Cultures were stained for (A–C) LAMP‐1 (D–E) LAMP‐2 or (F–G) LIMP‐II at the indicated time points. Representative A. LAMP‐1, (D) LAMP‐2 and F. LIMP‐II staining by confocal microscopy with enlarged image in insert. Number of intracellular bacteria per macrophage co‐localizing with (B–C) LAMP‐1, (E) LAMP‐2 and (G) LIMP‐II, three to four individual experiments performed in duplicate.*p < 0.05; **p < 0.01; ***p < 0.001, two‐way analysis of variance (ANOVA) with Sidak's post‐test. H. Differentiated THP‐1 macrophages were challenged with *S. pneumoniae* MOI = 5 for 6 h, treated with gentamicin, maintained up to 24 h and the number of intracellular bacteria co‐localizing with LIMP‐II estimated, three individual experiments in duplicate. **p < 0.01, two‐way ANOVA with Sidak's post‐test. I. Western blot of protein from mock infected (MI) or *S. pneumoniae* (Spn), *S. aureus* Newman (NEW) and USA300 (JE2) challenged differentiated THP‐1 cells 8 h post‐infection probed with anti‐cathepsin D (CatD) to record pro‐CatD (inactive) and the active heavy chain of catD, with actin used as loading control. J. The percentage expression of activate CatD heavy chain by densitometry relative to total CatD, and the figure above the active form represents the percentage of activation, three experiments, *p < 0.05, **p < 0.01, two‐way ANOVA with Sidak's post‐test.

We next addressed whether a defect in acidification was associated with maturation failure of the phagosome. Similar to internalization of other bacteria (Berger *et al*., 2010), including *S. pneumoniae* in macrophages (Gordon *et al*., 2000), the majority of *S. aureus* trafficked into a phagosome that acquired lysosomal‐associated membrane protein (LAMP)‐1 at all time points studied (Fig. 4B–C). The majority were also in phagolysosomes containing LAMP‐2 (Fig. 4E). Because LAMP‐1/2 are acquired after fusion with early endosomes and are required for fusion with late endosomes/lysosomes (Huynh *et al*., 2007), this suggested the initial steps in phagosomal maturation occurred normally. Next, we addressed if phagosomes containing *S. aureus* showed evidence of lysosomal fusion. The majority of *S. aureus*‐containing phagosomes failed to express the lysosomal marker lysosomal integral membrane protein (LIMP‐II (LGP85)) (Fig. 4G) (Kuronita *et al*., 2002; Huynh *et al*., 2007), in contrast to phagosomes containing *S. pneumoniae*, the majority of which appeared to mature into phagolysosomes as evidenced by acquisition of LIMP‐II (Fig. 4F). Impaired phagolysosomal maturation was also observed following exposure to a USA300 strain (Figure S8). A further feature of phagosomal maturation into a phagolysosome is its acquisition and activation of cathepsins, including cathepsin D (Flannagan *et al*., 2009). Recruitment of cathepsin D into a phagolysosome containing *S. pneumoniae* is associated with its activation (Bewley *et al*., 2011a), but in contrast to *S. pneumoniae*, we found little evidence of cathepsin D activation following phagocytosis of *S. aureus* (Newman or USA300 strains), which suggested it was not being recruited to an *S. aureus*‐containing phagolysosome or activated to a similar extent (Fig. 4I–J). Similar results were found with MDM (data not shown).

### Intracellular persistence of Staphylococcus aureus Newman involves ongoing cycles of macrophage lysis and bacterial re‐uptake

**Figure 5:**
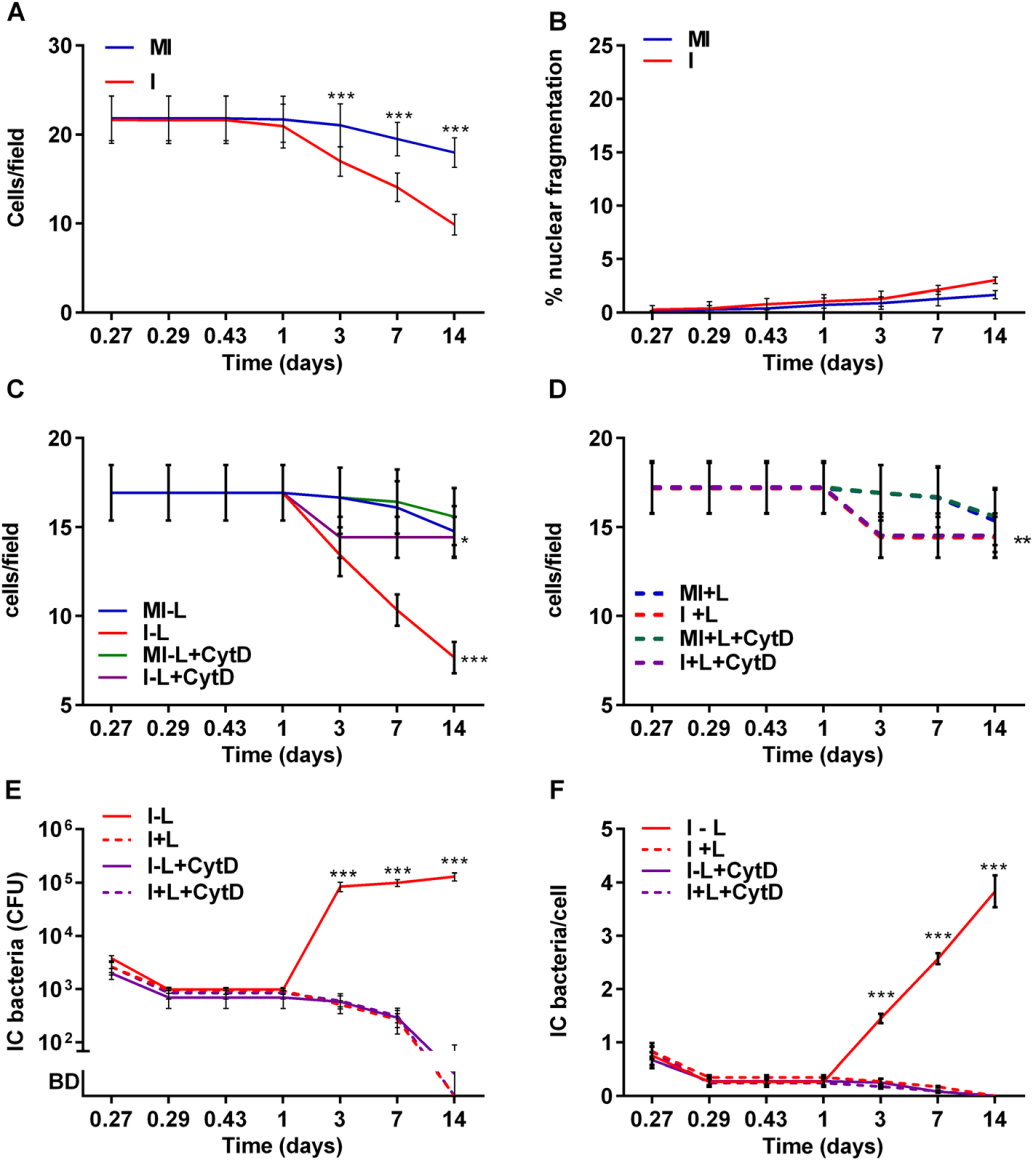
Macrophages maintain sustained viability following challenge with *S. aureus* Newman and continued macrophage lysis and re‐uptake of intracellular bacteria are required to maintain an intracellular pool of bacteria. Differentiated THP‐1 macrophages were mock infected (MI) or challenged with *S. aureus* Newman (I) MOI = 0.05 for up to 48 h. A. Macrophage viability measured as cells per field. B. Percentage of macrophages showing apoptotic nuclei indicative of apoptosis. The data represents three individual experiments performed in duplicate. *p < 0.05, ***p < 0.001, two‐way analysis of variance (ANOVA) with Sidak's post‐test versus mock infected at each time point. C–F. Cultures challenged with bacteria were incubated after 6 h in the presence (+) or absence (−) of low dose lysostaphin (L) or cytochalsin D (CytD). (C) Macrophage viability without lysostaphin ± CytD or D. with lysostaphin ± CytD. *p < 0.05 MI‐L + CytD versus I‐L + CytD, ***p < 0.001 MI‐L versus I‐L, I‐L versus MI‐L + CytD, and I‐L versus I‐L + CytD E. CFU of viable intracellular (IC) bacteria or F. number of intracellular bacteria per cell, three individual experiments were performed in duplicate.

Engagement of macrophage apoptosis after an extended period of intracellular phagolysosomal killing is a recognized host defence strategy that prevents intracellular persistence of micro‐organisms such as *S. pneumoniae* (Dockrell *et al*., 2001; Marriott *et al*., 2005). It is also dependent on cathepsin D activation in the bacterial‐containing phagolysosome (Bewley *et al*., 2011a). We measured cell viability for extended periods in macrophages cultured with *S. aureus* for up to 14 days. We found no evidence of early (in the first 24 h) loss of cell viability or of apoptosis (Fig. 5A–B), a finding also replicated with SH1000 (Figure S9A–B). In contrast, when macrophages were challenged with USA300, we observed increased loss of cell viability in the first 24 h and increased markers of loss of cell permeability (Figure S9C–E), in keeping with the propensity of this strain to express cytotoxic virulence factors such as the phenol soluble modulin PSMα3 at high levels and to cause neutrophil cell death (Li *et al*., 2010), but once again observed little apoptosis as demonstrated by caspase 3 activation or nuclear fragmentation (Figure S9F–G).

As shown in Fig. 5A, macrophage viability, however, declined from 3 to 14 days after exposure to bacteria. Consistent with some other reports (Jackson *et al*., 1995), the late cell loss of macrophages in the presence of extracellular bacteria was associated with only low levels of apoptosis (Fig. 5B). These findings of low rates of apoptosis were also replicated with SH1000 (Figure S9B) and USA300 (data not shown), and similar results were apparent with MDM (data not shown). The decline in cell numbers required ongoing bacterial phagocytosis and a pool of extracellular bacteria (Fig. 5C–D), while apoptosis remained low under all conditions (Figure S10A–B). Maintenance of a pool of viable intracellular bacteria also required sustained phagocytosis and a pool of extracellular bacteria (Fig. 5E–F). This suggested the intracellular pool of viable bacteria was not solely the result of intracellular replication in macrophages that had ingested bacteria in the early stages of the culture and remained viable but that ongoing bacterial uptake was required to support this late expansion in intracellular bacteria.

Because treatment with lysostaphin and cytochalasin D both depleted the intracellular reservoir, we hypothesized that the intracellular pool of bacteria was maintained by cycles of release from macrophages undergoing non‐apoptotic cytolytic cell death and further phagocytic uptake by the remaining viable macrophages. We tested this using video time lapse and found that while prolonged culture in lysostaphin prevented re‐emergence of the extracellular bacterial pool that in the presence of only a short pulse of lysostaphin, to kill extracellular bacteria, without prolonged culture the extracellular pool re‐emerged from an intracellular source (Figures S11–12, Video S1). Analysis of critical time points on a representative video between 52 and 63 h revealed that clumps of intracellular bacteria, presumably in an endosomal compartment, replicated and spread to fill the whole cytoplasm and were then released into the extracellular compartment where they further expanded. Analysis of the fluorescence intensity showed logarithmic expansion after cell lysis, consistent with logarithmic expansion of bacteria after release into the extracellular compartment (Figure S11B). We also observed that from 24 to 72 h after exposure to bacteria, macrophages appeared to lyse and release bacteria and that these bacteria were then taken up by further macrophages, explaining both the progressive loss of macrophages and the maintenance of an intracellular population. The rate of lyses suggested 3–5% of cells lysing/12 h period after 24 h. The video time lapse was also consistent with our prior experiments, because they provided no evidence of intracellular replication in the first 24 h after bacterial challenge; however, they suggested that at later time points from 24 to 72 h, when macrophages could no longer kill bacteria, there was intracellular bacterial replication, which proceeded macrophage lysis. Overall, these results showed that the late consequence of exhaustion of intracellular killing is intracellular replication, macrophage lysis and release of intracellular bacteria, which are then taken up by surviving macrophages whose killing capacity in turn becomes exhausted leading to further bouts of cells' lysis and bacterial release.

### *In vivo* *persistence of intracellular* *Staphylococcus aureus*

**Figure 6:**
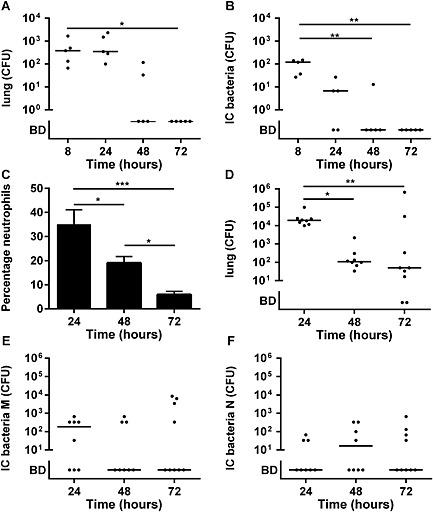
Mice demonstrate control of intracellular bacteria at low dose. (A–B) 10^5^ CFU of *S. aureus* were instilled into the lungs of mice. (A) CFU in the lungs and (B) intracellular **(**IC) CFU in bronchial alveolar macrophages 8, 24, 48 and 72 h post‐infection, *n* = 5. (C–F) Mice were instilled with 10^7^ CFU of *S. aureus* (C) neutrophil numbers in bronchoalveolar lavage (D) lungs CFU and IC CFU in bronchial alveolar (E) macrophages and (F) neutrophils 24 *n* = 8, 48 *n* = 8 and 72 h *n* = 9 post‐infection, *p < 0.05, **p < 0.01, analysis of variance with Dunn's multiple comparisons test.

Our results demonstrated a finite capacity of macrophages to kill intracellular *S. aureus* and that if this capacity was overwhelmed, intracellular bacteria could persist via a cycle involving cell lysis and re‐uptake. To examine whether bacteria persisted in macrophages *in vivo* in the lungs following low‐dose challenge or whether the macrophages had significant capacity to clear bacteria, we instilled low numbers of bacteria into the lungs of mice and lavaged alveolar macrophages at varying periods after infection. These experiments initially used low doses of bacteria, which were contained without any significant recruitment of neutrophils, because cytospins contained >95% alveolar macrophages and <3% neutrophils at all time points studied. Mice cleared bacteria from the lungs from 24 to 72 h after infection, but although 60% of mice had viable bacteria in their alveolar macrophages at 24 h and 20% at 48 h after infection, all mice had cleared intracellular bacteria from the lungs by 72 h (Fig. 6A–B). In these experiments, alveolar macrophage apoptosis remained low at all time points, with <5% apoptosis on cytospins, which was equivalent to mock‐infected samples (data not shown). This suggested intracellular bacteria could survive for limited periods in macrophages even following low dose infections, although they could be ultimately cleared from the lungs despite the limitations to macrophage‐mediated clearance. When we repeated experiments with higher doses of bacteria, which resulted in neutrophil recruitment to the lungs (Fig. 6C), we found that 78% of mice failed to clear bacteria from the lungs over a 72 h period (Fig. 6D). In keeping with this, we found persistent intracellular bacteria in 44% of the mice alveolar macrophages Fig. 6E (and also in their neutrophils; Fig. 6F), suggesting intracellular persistence in macrophages is an *in vivo* phenomenon when the capacity to control infection is overwhelmed.

## Discussion

As resident phagocytes, macrophages control the bacterial clearance of *S. aureus* from tissues, but how effectively macrophages perform this role is unclear. We demonstrate that differentiated macrophages have a limited capacity for intracellular killing of *S. aureus* and that sustained phagocytosis overwhelms intracellular killing. In contrast to several other extracellular bacteria, *S. aureus* are less likely to traffic to acidified phagosomes, and phagosomal maturation is incomplete. One consequence of this is a failure to engage a delayed programme of macrophage apoptosis‐associated killing. Bacteria persist as an intracellular pool, through extended waves of cell lysis and re‐uptake by macrophages.

Our understanding of the competence of tissue macrophages to affect bacterial clearance in comparison with that of recruited phagocytes such as neutrophils is incomplete. Tissue macrophages are adapted to their homeostatic roles, and the mechanisms used by differentiated macrophages to kill intracellular bacteria are incompletely defined. Much of our knowledge is based on inference, derived from observations involving the neutrophils, monocytes and undifferentiated macrophage cell lines. Our study provides an important advance by specifically studying differentiated macrophages, using both differentiated cell lines that effectively model innate immune responses in tissue macrophages (Daigneault *et al*., 2010) and also primary human macrophages. We confirm that at low MOI macrophages can kill almost all ingested *S. aureus*, but the capacity to up‐regulate killing is limited, and some intracellular bacteria have the potential to survive inside macrophages. IFN‐ γ, an effective stimulus for microbicidal strategies employed by macrophages, provided only a modest increment to intracellular killing and did not prevent intracellular persistence, showing there was little capacity to increase intracellular killing (Cassatella *et al*., 1985; Totemeyer *et al*., 2006). Surprisingly, phagocytosis was dissociated from intracellular killing capacity, and macrophages continued to accumulate further viable intracellular bacteria after the capacity for intracellular killing was overwhelmed.

We used DCF‐DA to estimate oxidant stress, recognizing results are also modified by nitrosative stress and cytochrome c release in apoptotic cells (Wardman, 2007) and therefore measuring production at an early time point when these other confounders are low (Marriott *et al*., 2004). Our results suggest that there are not major deficiencies in production of ROS in response to *S. aureus*, as compared with *S. pneumoniae*, and impaired ROS production does not explain bacterial persistence. Multiple microbicidal mechanisms combine to mediate intracellular killing, but bacteria block their activity (Flannagan *et al*., 2009). In *S. aureus*, the dehydrosqualene synthase, CrtM, facilitates oxidative stress resistance; an arginine deaminase is expressed similar to those that cause nitrosative stress resistance in other Gram‐positive bacteria, and extracellular adherence protein prevents neutrophil granule‐associated serine protease‐mediated killing (Degnan *et al*., 1998; Diep *et al*., 2006; Liu, 2009; Stapels *et al*., 2012). Thus, despite the generation of ROS and other microbicidals, additional antimicrobial mechanisms are required. Acidification of the phagosome containing *S. aureus* and activation of alternative mechanisms of intracellular bacterial killing are therefore critical. Microbial degradation in a mature acidified phagosome releases pattern recognition receptor ligands that mediate a second wave of TLR‐mediated pro‐inflammatory signals that help activate the macrophage response to *S. aureus* infection including generation of microbicidal molecules (Wolf *et al*., 2011). Prior studies have shown that ROS buffer the early steps in phagosomal acidification (Segal *et al*., 1981; Hampton *et al*., 1998) and that caspase 1 activation attempts to limit this buffering capacity in the early stages of the development of the *S. aureus*‐containing phagosome (Sokolovska *et al*., 2013). We demonstrate, however, that there is a sustained reduction in acidification of the phagosome by *S. aureus*, as compared with *E. coli* or *S. pneumoniae*. This reduction in acidification will compromise the intracellular killing of *S. aureus* and ultimately will lead to bacterial translocation from the phagolysosome (Kubica *et al*., 2008; Koziel *et al*., 2013). Our results suggest that before of any release there is a failure of acidification and appropriate maturation of the phagosome.

Failure to achieve optimal phagosomal acidification could arise because of dysfunction of hydrogen ion pumps such as the vacuolar ATPase or the Na^+^/H^+^ exchange (NHE1) found in the phagosomal membrane (Hackam *et al*., 1997). Impairment of phagosomal acidification also contributes to a failure of the phagosome to mature appropriately into a phagolysosome through disruption in endosomal trafficking (Gordon *et al*., 1980; van Deurs *et al*., 1996). Intracellular survival of bacteria is aided by impairing phagosomal acidification. *Mycobacterium tuberculosis* produces a protein tyrosine phosphatase (PtpA) that binds to a subunit of the vacuolar ATPase and blocks its trafficking and phagosomal acidification (Wong *et al*., 2011). Maturation of the phagosome follows a series of orderly events; the small GTPase Rab5A regulates fusion with early endosomes (Bucci *et al*., 1992), while Rab7A regulates their interaction with late endosomes and lysosomes (Harrison *et al*., 2003). LAMP‐1 and LAMP‐2 are recruited at a stage between the interaction with early and late endosomes (Huynh *et al*., 2007) and were recruited appropriately to *S. aureus*‐containing phagosomes. However, we observed that the phagosomes containing *S. aureus* had reduced levels of LIMP‐II, a marker of fusion with lysosomes (Kuronita *et al*., 2002). This suggested that a failure of acidification was associated with a failure of the later stages of phagolysosomal maturation.

A potential consequence of impaired phagolysosomal maturation is reduced activation of phagosomal proteases that contribute to microbial killing (Flannagan *et al*., 2009). Activation of cathepsin D is an upstream event that primes the macrophage to engage a programme of apoptosis‐associated bacterial killing, which is required to remove intracellular bacteria that canonical phagolysosomal microbicidal mechanisms have failed to kill (Bewley *et al*., 2011b; Bewley *et al*., 2011a; Aberdein *et al*., 2013). Cathepsin D is delivered to the phagosome by lysosomes, and a failure of phagolysosomal fusion results in decreased delivery of cathepsin D to the phagosome containing *S. aureu* *s* (Godbold *et al*., 1998). Moreover, impaired phagolysosomal acidification reduces the range of cathepsin D substrates, because its enzymatic range is related to pH (Capony *et al*., 1987). Cathepsin D activation plays a role in down‐regulating the anti‐apoptotic protein Mcl‐1, an essential regulator of apoptosis‐associated bacterial killing (Marriott *et al*., 2005; Bewley *et al*., 2011a). It is noteworthy that we and others have suggested that phagocytosis of *S. aureus* is associated with a failure to activate cathepsin D and with sustained up‐regulation of Mcl‐1, with the consequence that macrophage apoptosis does not occur (Bewley *et al*., 2011a; Koziel *et al*., 2013). Therefore, an important host defence strategy for persistent intracellular bacteria is not engaged.

We suggest that macrophages can provide an environmental niche in which *S. aureus* can survive, avoiding the immune system and many antimicrobials. Intracellular persistence of *S. aureus* has been described in macrophages and also non‐professional phagocytes such as epithelial cells, keratinocytes and endothelial cells (Vesga *et al*., 1996; von Eiff *et al*., 2001; Clement *et al*., 2005; Kubica *et al*., 2008). A variety of microbial factors have been associated with intracellular persistence including the alternative sigma factor B, the global regulator agr, alpha toxin and the ability to form small colony variants (Vesga *et al*., 1996; Kubica *et al*., 2008). Pore‐forming toxins such as alpha toxin could alter membrane integrity, compromising endosomal acidification, while phenol soluble modulin (PSM) α peptides are major factors mediating phagocyte cytolysis and facilitating phagosomal escape of bacteria in models where alpha toxin does not appear to be a significant cause of bacterial translocation into the cytoplasm (Grosz *et al*., 2014). We observed that a USA300 strain, associated with enhanced expression of PSM α peptides (Li *et al*., 2010), did not induce a greater degree of impairment of endosomal acidification, even though it did result in earlier cell cytotoxicity and a trend towards greater accumulation of intracellular bacteria in keeping with a greater disruption of macrophage function and of *S. aureus* killing. While these findings suggested strains expressing higher levels of PSM α peptides induce greater macrophage cytotoxicity, they also suggested the failure to acidify and appropriately mature phagolysosomes was more widespread amongst *S. aureus* strain and was not specifically associated with higher expression of PSM α peptides. Our findings are in agreement with those of Kubica who described intracellular survival of *S. aureus* in macrophages that did not undergo apoptosis or necrosis for several days but were ultimately lysed (Kubica *et al*., 2008). We extend these observations by showing that these persistent bacteria are the result of an inability to kill all ingested bacteria and that persistence is associated with a failure of phagosomal maturation. Our data suggest that viable intracellular *S. aureus* will overwhelm host defence and, through cycles of uptake and release, will be maintained for sustained periods of time, as we show can happen *in vivo* when we push the infecting dose of *S. aureus* in the lungs to high levels, above the capacity of intrinsic innate host defence mechanisms to mediate clearance.

In conclusion, we demonstrate that although macrophages readily phagocytose *S. aureus*, macrophages' capacity for intracellular killing is rate‐limiting for bacterial clearance. A failure of phagosomal maturation and acidification is associated with an absence of apoptosis‐associated bacterial killing in macrophages. Viable bacteria accumulate because of ongoing phagocytosis and form an intracellular pool that is maintained through cycles of cell lysis and phagocytosis by other macrophages. Targeting this population of bacteria should be a focus of future studies in an effort to limit the relapsing and metastatic capacity of *S. aureus* infections.

## Experimental procedures

### Cell culture and differentiation

The human monocytic leukaemia cell line THP‐1 was obtained from ATCC and maintained in RPMI 1640 medium (Lonza) supplemented with 10% of low endotoxin heat inactivated foetal calf serum (HIFCS, Hyclone) and 2 mmol l^−1^ of l‐glutamine (Sigma). THP‐1 cells were differentiated using 200 nM of phorbol‐12‐myristate 13‐acetate (PMA) (Sigma) for 3 days followed by 5 days rest as outlined previously (Daigneault *et al*., 2010). Cells were seeded at 2 × 10^5^ cells/ml (1 ml/well), which results in 2 × 10^5^ cells/well at the time of infection. MDMs were isolated from peripheral blood mononuclear cells from healthy donors, as previously described (Dockrell *et al*., 2001) with ethical approval from the South Sheffield Research Ethics Committee (07/Q2305/7) (Dockrell *et al*., 2001; Daigneault *et al*., 2010). Peripheral blood mononuclear cells were isolated by Ficoll Plaque (GE healthcare) density centrifugation seeded at 2 × 10^6^ cells/ml in RPMI 1640 medium supplemented with 10% of newborn foetal calf serum (Fischer) in 24 well plates (Corning) with 1 ml/well to give approximately 2 × 10^5^ MDM/well. After 24 h, non‐adherent cells were removed, and adherent cells were cultured in RPMI 1640 medium with 10% of low endotoxin HIFCS and 2 mmol l^−1^ of l‐glutamine and used for 14 days. Representative wells were scraped at the time of infection to confirm cell number for calculation of MOI.

### Bacterial strains

All experiments were performed with *S. aureus* Newman strain unless otherwise stated. All strains used, with the exception of *S. aureus* Newman tagged with green fluorescent protein (GFP), were sourced from Professor S. Foster at the University of Sheffield. *S. aureus* Newman strains were grown in brain–heart infusion (BHI) medium (Sigma) to OD_600nm_ 0.6, and SH1000 and JE2 strains were grown to OD_600nm_ 1.0. A kanamycin‐resistant Newman *S. aureus* strain (Kan^R^) was grown in BHI medium supplemented with 50 µg ml^−1^ of kanamycin (Sigma). *S. aureus* Newman GFP was prepared by phage transduction. Briefly, *S. aureus* Newman was streaked onto a modified Luria–Bertani plate containing 7 g l^−1^ of potassium chloride (KCl) (Sigma), designated LK media, and left at 37 °C for 16 h then stored at 4 °C. Ten colonies were inoculated into 50 ml of LK broth and grown for 16 h at 37 °C on a shaker then centrifuged at 5000 g for 10 min at room temperature (RT). The pellet was resuspended in 3 ml of LK broth and then 500 µl of *S. aureus* Newman recipient cells, 1 ml of LK broth, 10 µl of 1 M of calcium chloride (Sigma) and 500 µl of phage lysate was combined and incubated at 37 °C for 25 min and then for a further 15 min on a shaker. One millilitre of ice cold 0.02M sodium citrate (Sigma) was added and left for 5 min at 4 °C and then centrifuged at 5000 g for 10 min at 4 °C. The pellet was resuspended in 1 ml of ice cold 0.02 M of sodium citrate and incubated at 4 °C for 1 h. One hundred microlitres were streaked onto selective plates (LK plus 5 µg ml^−1^ of tetracycline (Sigma) and 5 µg ml^−1^ of citrate (Sigma)), incubated at 37 °C for 16 h and then resubcultured the next day. *E. coli* strain C29, group 2 capsular serotype K54, was grown in BHI medium to mid‐log phase (Webster *et al*., 2010), *S. pneumoniae* D39 was grown as previously described (Dockrell *et al*., 2003). *S. aureus and S. pneumoniae* were stored in frozen aliquots at −80 °C and thawed before infection. Viable counts were determined after thawing, using the surface viable count method on blood agar (Miles *et al*., 1938).

### Macrophage bacterial challenge

Differentiated macrophages were challenged with *S. aureus* at various MOI. *S. aureus* was thawed, washed in phosphate buffered saline (PBS), added to the macrophages in the presence of fresh media, incubated on ice for 1 h and then at 37 °C, 5% of CO_2_. In some experiments, macrophages were fixed in 2% of paraformaldehyde (PFA) at 4 °C for 15 min and washed with PBS before challenge with bacteria. In experiments involving prolonged culture of macrophages, extracellular bacteria were killed at 6 h by the addition of 20 µg ml^−1^ of lysostaphin (Biosynexus) for 30 min (Schindler and Schuhardt, 1964) and then cells cultured up to 48 h in media containing 2 µg ml^−1^ of lysostaphin. After this period, cultures were subsequently incubated in the presence or absence of 2 µg ml^−1^ of lysostaphin. In certain experiments, 5 μM cytochalasin D (Sigma) was added to inhibit phagocytosis (DeLoid *et al*., 2009), or 50 μM Trolox (Sigma) was added to inhibit ROS. Reagents were added 30 min before infection and following any media change. In other experiments, macrophages were stimulated with 50 ng ml^−1^ of IFN‐γ (Sigma) for 18 h before challenge with *S. aureus* to enhance intracellular killing. Challenge with *E. coli* or *S. pneumoniae* was similar to that with *S. aureus* except that *S. pneumoniae* were first opsonized in immune serum as previously described (Dockrell *et al*., 2001). Extracellular colony forming units (CFU) were quantified by surface viable count on blood agar (Miles *et al*., 1938).

### Quantification of viable intracellular bacteria

To estimate intracellular bacterial load, macrophages were washed in PBS, fresh media containing 20 µg ml^−1^ of lysostaphin were added, and cultures were incubated at 37 °C, 5% of CO_2_ for 30 min (Schindler and Schuhardt, 1964). Cells were washed in PBS and incubated with 2% of saponin (Sigma) at 37 °C for 12 min. PBS was added to the cells; they were lysed by vigorous pipetting, and the number of viable intracellular bacteria was determined by surface viable count (Dockrell *et al*., 2001). To confirm complete killing of extracellular bacteria, some wells were fixed in 2% of PFA before challenge with bacteria and then exposed to lysostaphin and lysed as mentioned earlier, showing absence of bacteria in lysates. To estimate the kinetics of intracellular killing after the initial bacterial challenge (‘pulse’), extracellular bacteria were killed with lysostaphin treatment as mentioned earlier, and cells were cultured in fresh media with 2 µg ml^−1^ of lysostaphin (‘chase’) for 0.5‐4 h prior to lysis and intracellular bacterial quantification. Key experiments were repeated using gentamicin 20 µg ml^−1^ to kill extracellular bacteria and then culture in media‐containing vancomycin (0.75 µg ml^−1^; Sigma) during the ‘chase’ phase, with similar results (data not shown).

To test the contribution of ongoing ingestion to intracellular bacterial accumulation, the initial ‘pulse’ was terminated by incubation in the presence of 50 µg ml^−1^ of kanamycin at 37 °C for 30 min, and some cells were lysed for quantification of intracellular bacteria. The remaining macrophages were either maintained in 2 µg ml^−1^ of lysostaphin and subsequently lysed as per the original ‘pulse‐chase’ design, −Kan^R^ cultures (Kan^R^−), or were challenged with *S. aureus* Kan^R^ for 5–7 h; extracellular bacteria killed with 20 µg ml^−1^ of lysostaphin; and cells lysed as mentioned earlier, designated Kan^R^+ cultures (Kan^R^+). Some cells were treated with 2% of PFA before the second ‘pulse’ of bacteria to confirm all bacteria in lysates were the result of internalization. Total intracellular CFU bacteria were quantified by culture in blood agar (Total CFU) and intracellular *S. aureus* Kan^R^ quantified by growth in blood agar containing 50 µg ml^−1^ of kanamycin to determine the *S. aureus* Kan^R^ CFU. Levels of the CFU of *S. aureus* Kan^S^ (wild‐type; WT) were estimated in the Kan^R^+ cultures as Total − Kan^R^ CFU (i.e. the Kan^R^ CFU was subtracted from total CFU), and the values of Kan^S^ showed good agreement with the cultures treated without a second pulse of *S. aureus* Kan^R^ (Kan^R^− cultures).

### Live and dead staining

Macrophages were challenged with live *S. aureus*, or *S. aureus* that were heat killed at 80 °C for 15 min (as a control), at an MOI of 0.05 or 5 for 5 h. Extracellular bacteria were killed; cultures were treated with 2% of saponin and lysed as mentioned earlier. Supernatants were centrifuged at 3300 g for 30 s and then at 100 *g* for 8 min. Supernatants were then either left unstained or stained with 3 μM of DRAQ7 (Cell Signalling) for 10 min at 4 °C, and fluorescence was measured on the flow cytometer (LSRII, BD bioscience) using the red 660/20 nm laser, with at least 10 000 events captured and analysed using the BD Biosciences FACS Diva Software (version 8.0). DRAQ7 low events were recorded as non‐viable bacteria.

### Measurement of intracellular ROS

Macrophages were challenged with *S. aureus*, *S. pneumoniae* or mock infected, and extracellular bacteria were killed with lysostaphin (*S. aureus*) or gentamicin (*S. pneumoniae*). Macrophages were incubated in fresh media with or without 20 μM of DCF‐DA for 30 min at 37 °C, detached and analysed by flow cytometry.

### Fluorescence microscopy

For fluorescence microscopy, macrophages were seeded as mentioned earlier in 24 well plates (Corning) containing glass coverslips (Mensel‐Glazer). Macrophages were incubated with *S. aureus* or other bacteria as indicated for varying time points and then fixed in 2% of PFA for 15 min at 4 °C. Macrophages were washed with PBS, incubated with 3% of bovine serum albumin (Biowhittaker) for 30 min at RT, washed and incubated with an anti‐staphylococcal (rabbit) polyclonal IgG primary antibody (Zytomed Systems, 619 0198) at 1 : 1000 dilution for 10 min at RT. Cells were then washed and incubated with 1 : 250 of goat anti‐rabbit Alexa Fluor 568 secondary antibody (Invitrogen) for 10 min at RT, with cultures incubated with secondary alone serving as a control. To stain bacteria in endosomes, cultures were washed and incubated overnight with 0.01% of saponin to permeabilize cells (Jamur and Oliver, 2010) in the presence of antibodies against LAMP‐1 (mouse IgG1 monoclonal, clone H4A3 ab25630, Abcam), LAMP‐2 (mouse IgG1 monoclonal, clone H4B4 ab25631, LIMP‐II/(LGP85) (rabbit IgG polyclonal, Abcam, ab183856) all at 1 : 100 at 4 °C in the dark. Cells were washed and incubated with goat anti‐mouse Alexa Fluor 488 secondary antibody (Invitrogen) or in the case of LIMP‐II with goat anti‐rabbit Alexa Fluor 568 secondary antibody (Invitrogen), all at 1 : 250 for 90 min at RT in the dark. Cultures were washed and mounted on slides using Vectashield mounting medium containing 4′,6‐diamidino‐2‐phenylindole (DAPI) (Vector Laboratories) and visualized using the triple filter on the Leica DMRB fluorescent microscope using the ×100 objective. In certain experiments, images were obtained using a multiphoton confocal laser scanning microscope at ×63 oil immersion lens (Zeiss LSM510 NLO Inverted). One hundred macrophages were counted per sample and scored for adherent bacteria (DAPI positive and Alexa Fluor 568 positive in the presence of anti‐staphylococcal antibody), intracellular bacteria (DAPI positive, Alexa Fluor 568 negative) and intracellular bacteria colocalizing with LAMP‐1 or LAMP‐2 (DAPI positive and Alexa Fluor 488 positive, Alexa Fluor 568 negative if anti‐staphylococcal antibody added) and LIMP‐II (DAPI positive, Alexa Fluor 568 positive in the presence of anti‐LIMP‐II antibody). In select experiments, as indicated in individual figure legends, sequential staining of extracellular bacteria and LAMP‐1 was performed as mentioned earlier. To analyse cell number and nuclear morphological changes consistent with apoptosis, macrophages were stained with DAPI and analysed as described previously (Dockrell *et al*., 2001).

### Staining of bacteria with pHrodo

*Staphylococcus aureus*, *S. pneumoniae* or *E. coli* were incubated with 10.2 μM of pHrodo (Invitrogen) at 37 °C, with shaking in the dark for 30 min. The bacteria were centrifuged at 9300 g for 2 min (1 min in the case of *S. aureus*), and the bacterial pellet was washed and resuspended in PBS. *S. pneumoniae* was opsonized in RPMI with 10% v/v of anti‐pneumococcal immune serum prior to staining. Macrophages were challenged with bacteria as mentioned earlier and fixed in 0.2% of PFA for 30 min at RT. One hundred macrophages were counted, and the number of pHrodo positive/negative bacteria per macrophage was counted. pHrodo positive bacteria were taken to be in a compartment of pH 4–6, whereas pHrodo negative bacteria were taken to be in a compartment of pH > 6 in keeping with the fluorescence spectrum of the dye. To confirm the pH at which pHrodo was fluorescing, *S. aureus* was incubated with the dye and then fixed in 2% of PFA at RT for 15 min. The bacteria were then placed in PBS of different pH (4, 5, 6, 7 and 8) and images were taken using the ×63 lens of the Zeiss LSM 510 NLO Inverted Microscope. Fluorescence intensity was quantified using ImageJ and normalized against DAPI fluorescence (Figure S5). The pHrodo fluorescence intensity was also measured by adding labelled bacteria to a 96 well plate and measuring fluorescent intensity in a fluorescent plate reader normalizing to the OD_600_ of each well.

### Video time lapse microscopy

Macrophages were challenged with *S. aureus* Newman‐GFP strain at an MOI of 5 for 6 h or mock infected, and extracellular bacteria were killed with lysostaphin. Macrophages were then incubated in fresh media without sodium hydrogen carbonate with or without lysostaphin for up to 72 h. Imaging was started at 52 h post infection, imaging every 10 min until 72 h. Images were taken using the ×30 DIC/GFP laser on the Nikon Ti inverted fluorescence microscope with a 20 × 0.75 NA lambda objective lens. Images were captured with Neo camera (Andor) using NIS Elements (Nikon). The microscope was enclosed in temperature‐controlled and humidity‐controlled cabinet (OKO Labs) maintained at 37 °C. Images were taken from four fields of view from three random wells and analysed using the NIS Elements Viewer software version 4.20 (Nikon).

### Lactate dehydrogenase assay

The release of lactate dehydrogenase was measured using the Cytotox 96 cell viability kit (Promega), used according to the manufacturer's instructions (Bewley *et al*., 2014).

### Apoptosis and necrosis in live cells

Caspase activity in live cells was measured using the CellEvent caspase 3/7 green flow kit (Life Technologies), according to the manufacturer's instructions. Cells were co‐stained with TOPRO‐3 as a marker of necrosis, and cells were analysed by flow cytometry.

### Measurement of active cathepsin D

Differentiated THP‐1 cells grown in T25 flasks (2 × 10^6^ cells/flask) were challenged with *S. aureus* (MOI = 5), *S. pneumoniae* (MOI = 10) or mock infected as mentioned earlier and lysed for total protein 8 h post‐challenge as described previously (Dockrell *et al*., 2001; Marriott *et al*., 2005). Proteins were subject to SDS‐PAGE and Western immunoblotting, and membranes were probed with antibodies against cathepsin D (R and D systems, goat polyclonal, 1 : 1000), detecting pro‐cathepsin D/processing intermediates at approximately 46–51 kDa, and the heavy chain of active cathepsin D at approximately 34 kDa (Abcam), Mouse monoclononal (CTD‐19, 1 : 1000)], with the active cathepsin D band at 34 kDa, and actin as loading control (Sigma‐Aldrich rabbit polyclonal, 1 : 2000) as previously described (Bewley *et al*., 2011a).

### In vivo *model*

Female C57/Bl6 mice were instilled with 10^5^ or 10^7^ CFU of *S. aureus* Newman strain by intratracheal instillation as described previously (Dockrell *et al*., 2003). The 24, 48 and 72 h post‐infection mice were killed by overdose of pentabarbitone, lungs lavaged with 4 × 1 ml aliquots of saline for bronchial alveolar lavage (BAL) and lungs collected. BAL from mice instilled with 10^5^ CFU of *S. aureus* was centrifuged at 1000 g for 5 min; the cell pellet was incubated with 2% of saponin for 12 min and counts of viable bacteria were performed on lysates as mentioned earlier. BAL from mice instilled with 10^7^ CFU of *S. aureus* was centrifuged at 1000 g for 5 min; the cell pellet was resuspended in 1 ml of DMEM + 10% of HIFCS and 100 U/ml of penicillin; 200 µl was used for cytospins for differential cell count, and the rest was cultured in 24 well plates to allow adhesion of macrophages. The tissue culture medium containing non‐adherent cells (neutrophil fraction) was removed after 2 h and the cells were pelleted and lysed, and counts of viable bacteria were performed on the cell pellet and adherent macrophages as mentioned earlier. Lungs were homogenized, and viable bacteria were measured (Dockrell *et al*., 2003). All animal experiments were performed in accordance with the UK Animals (scientific procedures) Act 1986, authorized under a UK Home Office License and approved by the animal Ethical Review Committee of the University of Sheffield.

## Statistics

All graphs are represented as mean ± SEM unless otherwise stated. Statistical analysis was performed using Graphpad Prism® version 6.02. Statistical significance was determined as *p* < 0.05. All statistical tests used are listed in the figure legends.

## Conflict of interest

The authors declare that they have no conflict of interest.

## Supporting information


**Fig. S1.** Macrophages demonstrate accumulation of viable bacteria with increasing MOI.
**Fig. S2.** Macrophage exhaustion of initial killing following phagocytosis is not inoculum, macrophage type or strain dependent.
**Fig. S3.** Macrophage accumulation of intracellular bacteria is not the result of intracellular replication.
**Fig. S4.** Reactive oxygen species generation following macrophage challenge with bacteria.
**Fig. S5.** pHrodo labelled S. aureus fluoresces at low pH.
**Fig. S6.** Failure of intracellular S. aureus to traffic to an acidified endosome is not time or dose dependent.
**Fig. S7.** Failure of intracellular S. aureus USA300 to traffic to an acidified endosome is not time or dose dependent.
**Fig. S8.** S. aureus USA300 JE2 traffic to endosomes which demonstrate incomplete maturation.
**Fig. S9.** Macrophage apoptosis is not engaged with S. aureus.
**Fig. S10.** Low macrophage apoptosis is not affected by blocking phagocytosis and killing extracellular bacteria.
**Fig. S11.** With prolonged culture viable intracellular bacteria replicate and induce macrophage lysis.
**Fig. S12.** Intracellular replication occurs before lysis.

Supporting info item

Supporting info item

Supporting info item
