# Supplementary material for: Inability to sustain intraphagolysosomal killing of Staphylococcus aureus predisposes to bacterial persistence in macrophages
Source: Cell Microbiol. 2015 Sep 2;18(1):80–96. doi: 10.1111/cmi.12485 (PMC4778410; doi:10.1111/cmi.12485)
Supplement: Supplementary file 1 — Supporting info item [file CMI-18-80-s001.pdf]

Supporting Material

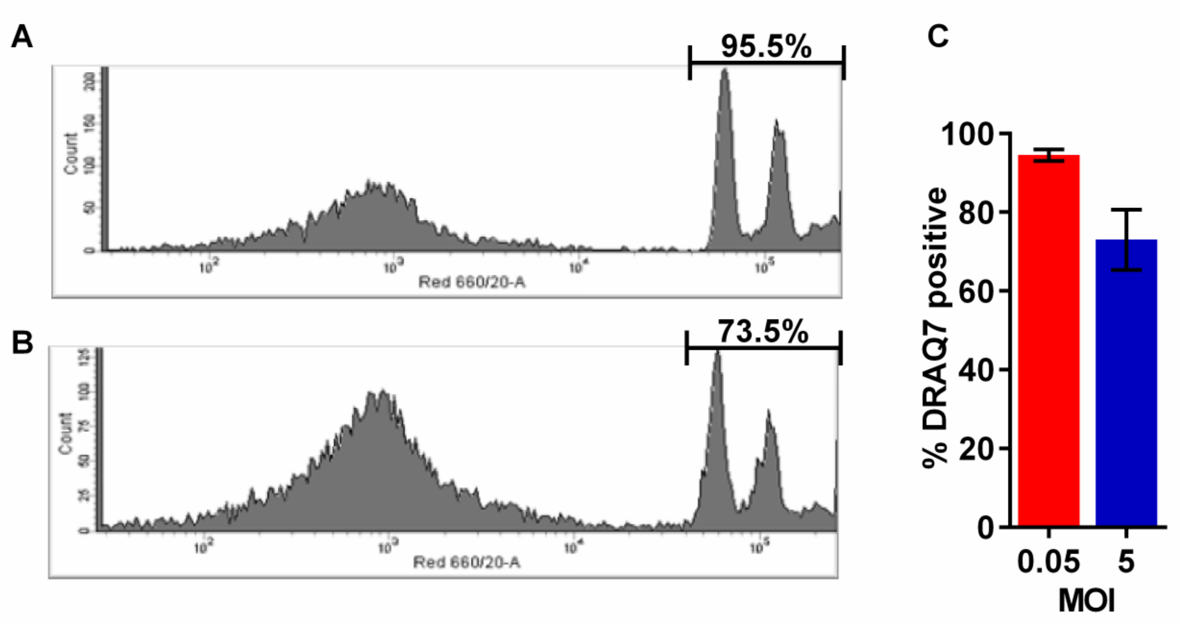

Figure S1: Macrophages demonstrate accumulation of viable bacteria with increasing MOI. Differentiated THP-1 macrophages were challenged with *S. aureus* Newman at an MOI of 0.05 or 5 for 5 h. Cultures were treated with lysostaphin and then lysed to recover intracellular bacteria and determine bacterial viability with DRAQ7. **(A)** Representative histogram at MOI of 0.05, **(B)** Representative histogram at MOI of 5. The histograms represent one representative experiment from the three individual experiments performed. **(C)** Percentage of intracellular non viable bacteria at MOI 0.05 and MOI 5, 3 individual experiments.

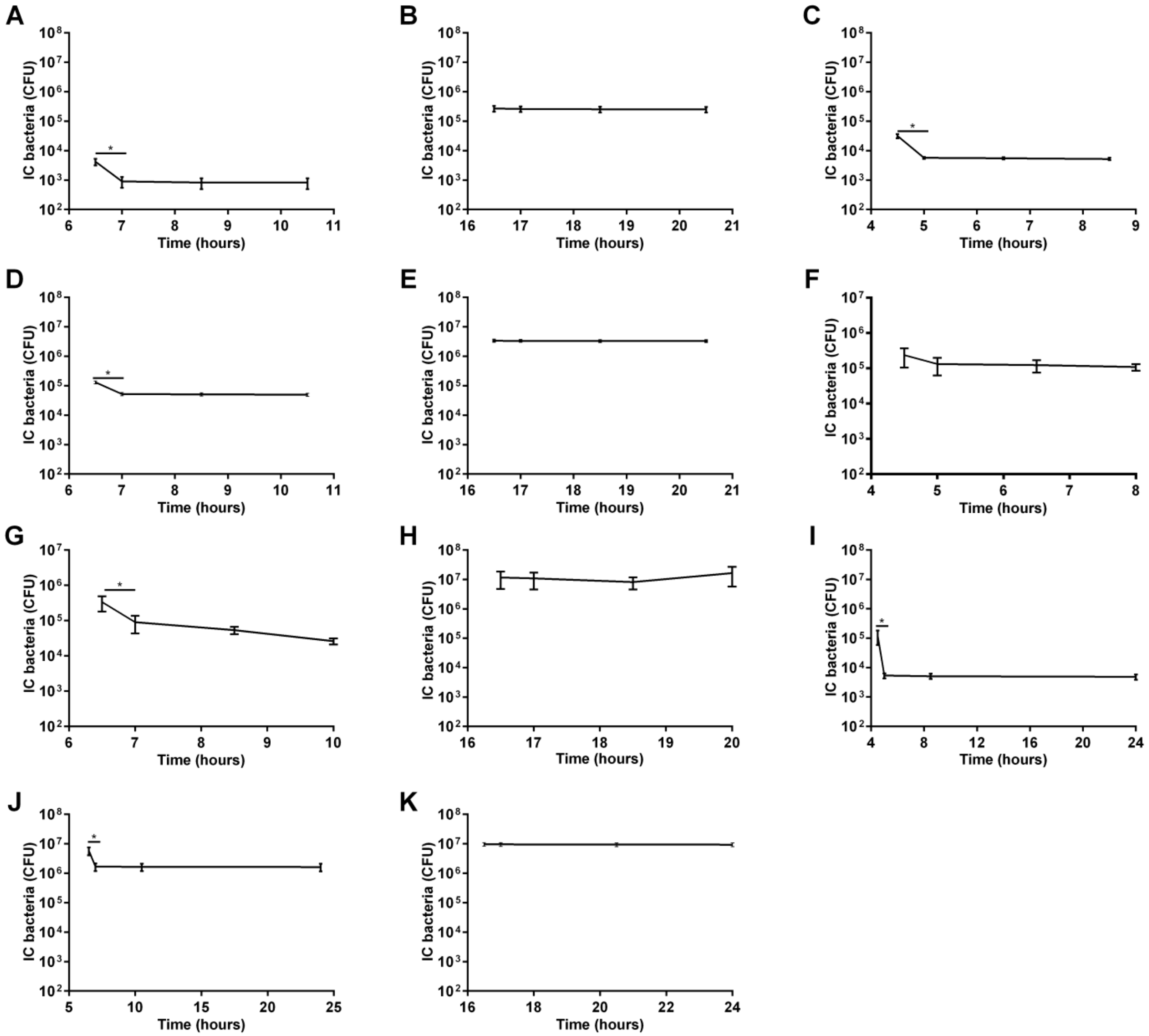

Figure S2: Macrophage exhaustion of initial killing following phagocytosis is not inoculum, macrophage type or strain dependent. Differentiated THP-1 macrophages were challenged with **(A-B)** *S. aureus* Newman MOI=0.05 for **(A)** 6 h, **(B)** 16 h, **(C-E)** *S. aureus* SH1000 MOI=5 for **(C)** 4 h, **(D)** 6 h, **(E)** 16 h or **(F-H)** *S. aureus* JE2 MOI=5 for **(F)** 4 h, **(G)** 6 h, **(H)** 16 h. **(I-K)** Monocyte-derived macrophages were challenged with *S. aureus* Newman MOI=5 for **(I)** 4h. **(J)** 6h, **(K)** 16h. Cultures were treated with lysostaphin and then maintained in low dose lysostaphin until lysed at the indicated time points for intracellular (IC) CFU quantification. *p<0.05 repeated measures ANOVA with Sidak's multiple comparisons post-test comparing the first two time points, 3 individual experiments.

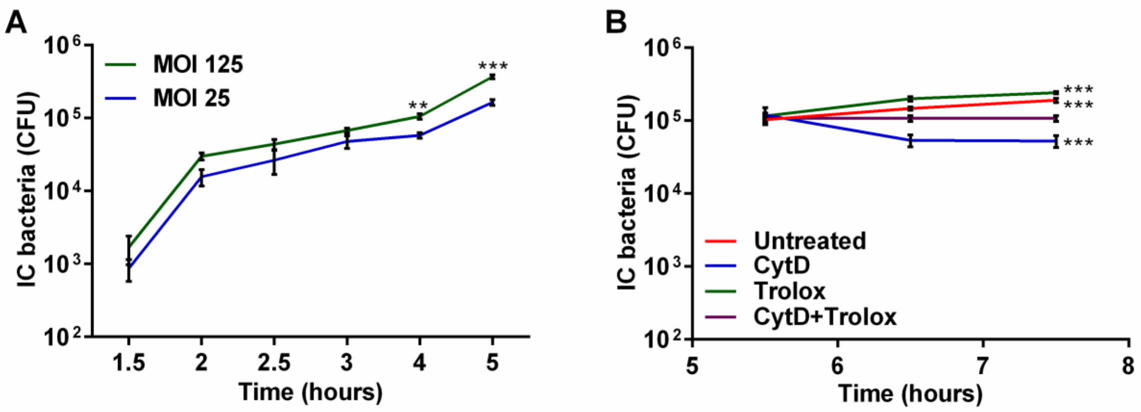

Figure S3: Macrophage accumulation of intracellular bacteria is not the result of intracellular replication. Differentiated THP-1 macrophages were **(A)** challenged with *S. aureus* Newman MOI=25 or 125 for 1.5-5 h and lysed for intracellular (IC) CFU quantification, 3 independent experiments. **p<0.01, ***p<0.001, Two Way ANOVA with Sidak's multiple comparisons test comparing MOI=25 and 125 at each time point. **(B)** Differentiated THP-1 macrophages were challenged with *S. aureus* at an MOI of 5 for 4 h and then treated with vehicle, or incubated with cytochalasin D, Trolox or both. Cultures were lysed at the indicated time points for intracellular CFU quantification, 3 individual experiments. ***p<0.001, Two Way ANOVA with Dunnett's multiple comparisons test comparing each treatment with untreated at 7.5 h.

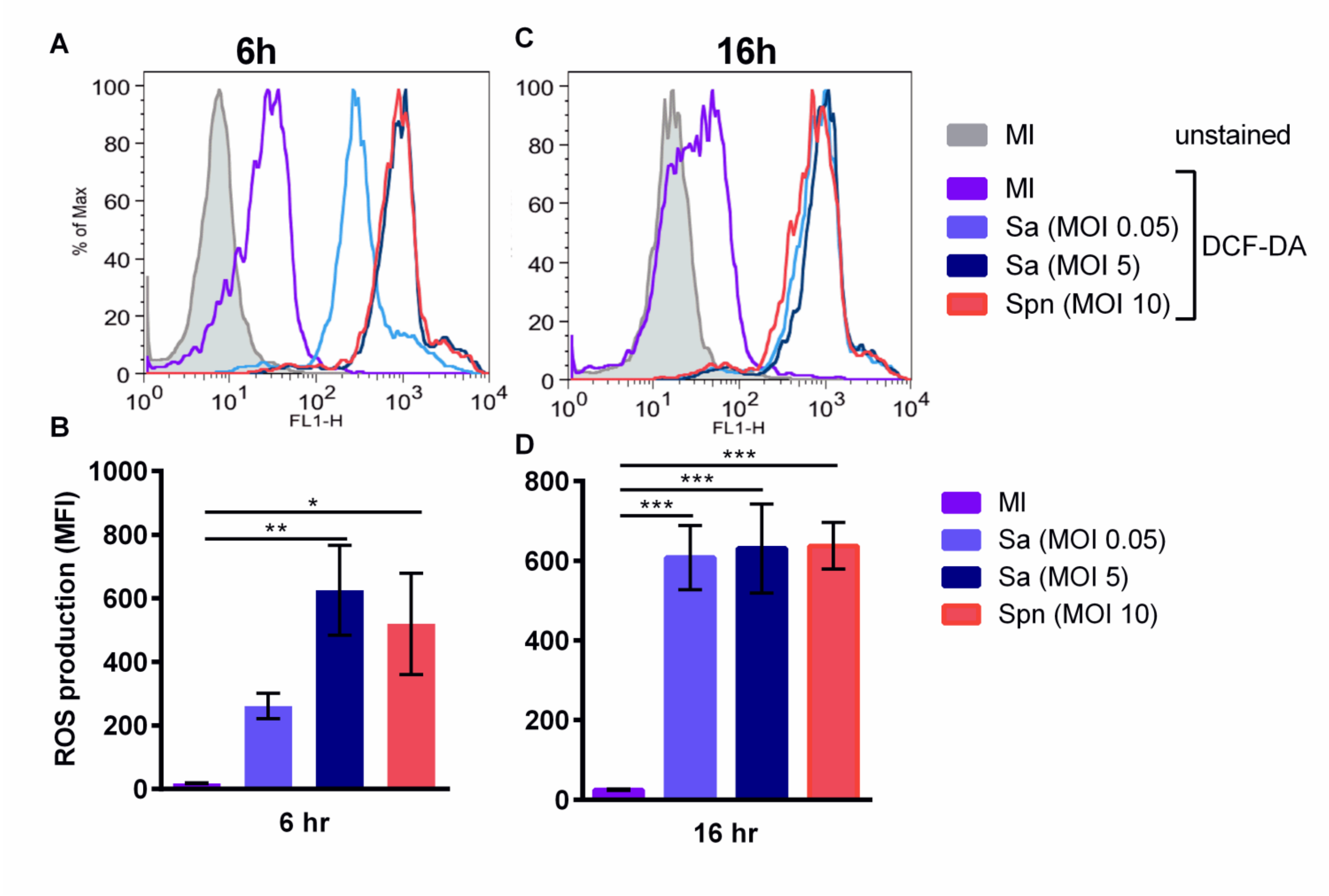

Figure S4: Reactive oxygen species generation following macrophage challenge with bacteria. Differentiated THP-1 macrophages were mock-infected (MI) or challenged with *S. aureus* Newman MOI of 0.5 and 5, or opsonized *S. pneumoniae* MOI of 10 for 6 h or 16 h and reactive oxygen species generation measured using 2', 7'–dichlorofluorescin diacetate (DCF-DA). **(A, C)** Representative histograms at **(A)** 6 h and **(C)** 16 h **(B, D)** Median fluorescent intensity (MFI) of DCF-DA **(B)** 6 h and **(D)** 16 h, three independent experiments *p<0.05, ***p<0.001, Two Way ANOVA with Dunnett's Post Test *versus* untreated.

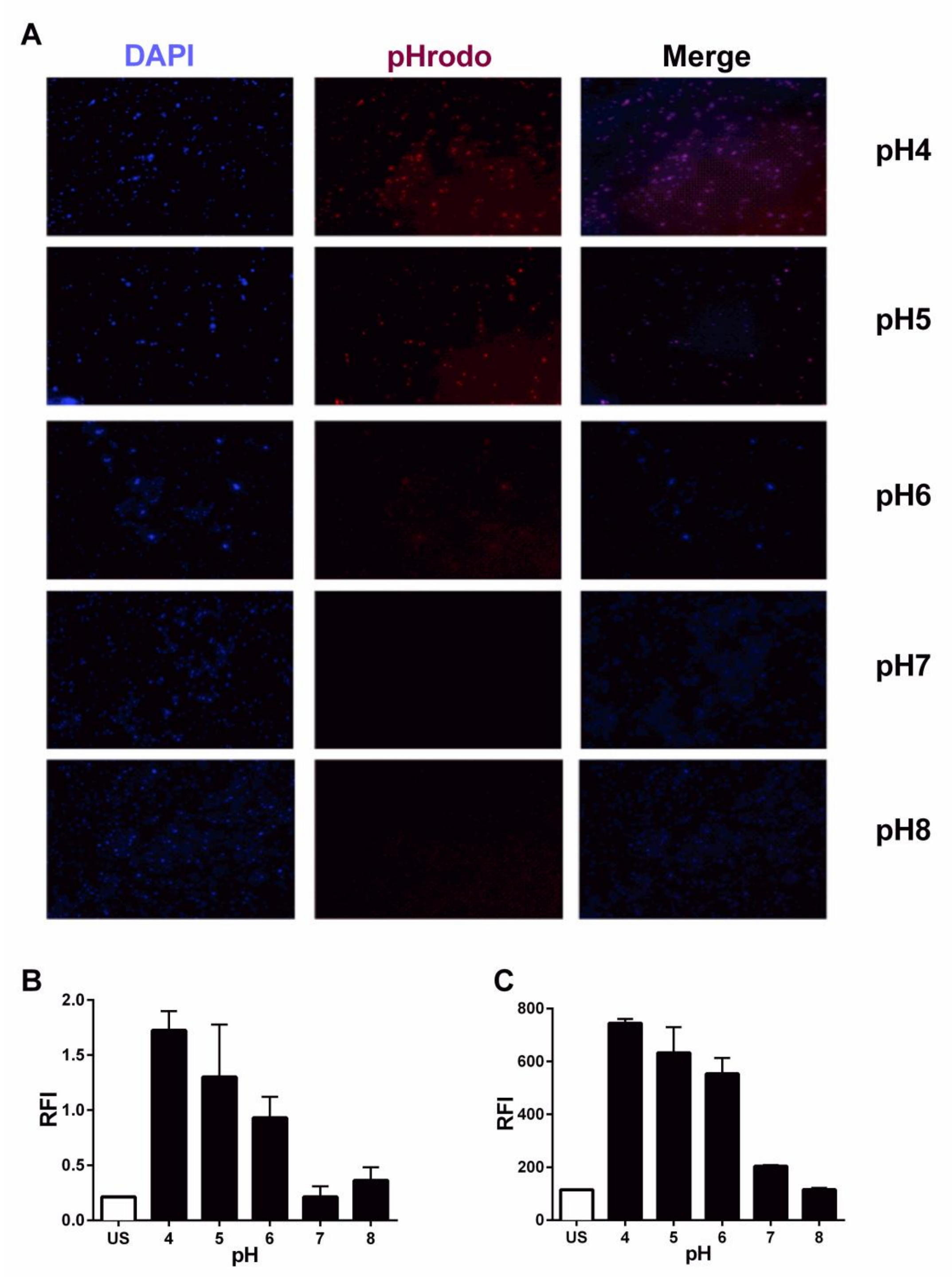

Figure S5: pHrodo labelled *S. aureus* fluoresces at low pH. *S. aureus Newman* was labelled with pHrodo and then fixed and incubated in PBS of different pHs. **(A)** Representative images and **(B)** relative fluorescent intensity (RFI) of images normalised to DAPI using ImageJ. **(C)** RFI of pHrodo labelled *S. aureus* as measured in a fluorescent plate reader), n=3.

.

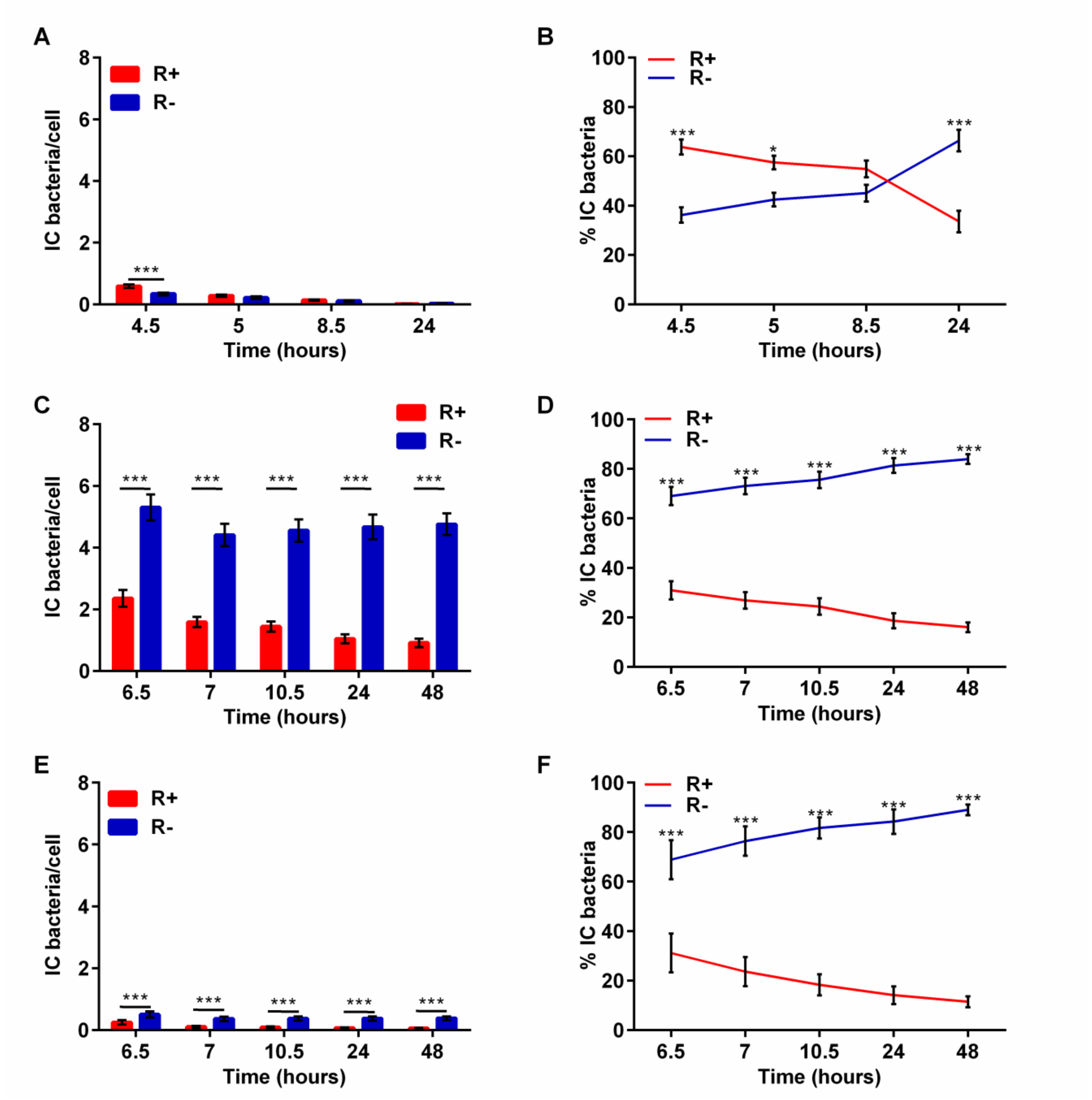

Figure S6: Failure of intracellular *S. aureus* to traffic to an acidified endosome is not time or dose dependent. Differentiated THP-1 macrophages were challenged with pHrodo labelled *E. coli* MOI=5 for 4 h. Cultures were treated with gentamicin and then maintained in low dose gentamicin until analyzed **(A)** Number of intracellular pHrodo fluorescent (R+) or non-fluorescent (R-) *E. coli* and **(B)** percentage of intracellular R+/R- *E. coli*. Differentiated THP-1 macrophages were challenged with *S. aureus* Newman labelled with pHrodo at an MOI of 5 **(C-D)** or 0.05 **(E-F)** for 6 h then treated with lysostaphin and maintained in low dose lysostaphin until analyzed, **(C, E)** number and **(D, F)** percentage of intracellular *S. aureus* colocalising with pHrodo after 6.5-48 h, 3 individual experiments performed in duplicate. *p<0.05, **p<0.01, ***p<0.001, Two Way ANOVA with Sidak's Post Test R+ *vs.* R-.

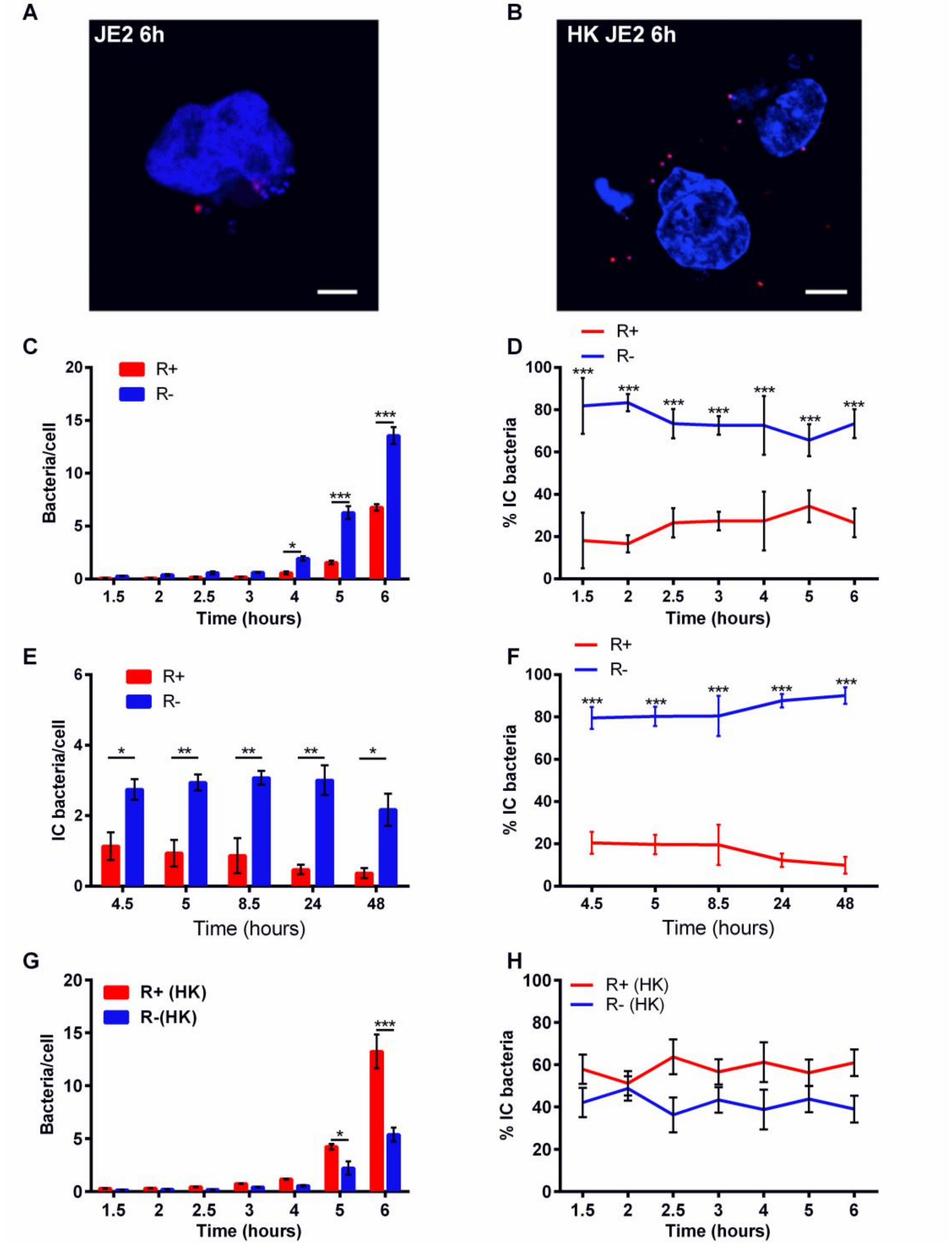

Figure S7: Failure of intracellular *S. aureus* USA300 to traffic to an acidified endosome is not time or dose dependent. Differentiated THP-1 macrophages were challenged with pHrodo labelled *S. aureus* USA300 strain JE2, MOI=5 for the indicated times. The extended 4.5-48 h cultures were treated with gentamicin and then maintained in low dose gentamicin until analyzed. Representative image of internalized **(A)** JE and **(B)** heat killed JE2. Cultures were analyzed from 1.5-6 h **(C-D)** or 4.5-48 h **(E-F).** **(C, E)** Number of intracellular pHrodo fluorescent (R+) or non-fluorescent (R-) JE2 and **(B, D)** percentage of intracellular R+/R- JE2, 3 individual experiments performed in duplicate. *p<0.05, **p<0.01, ***p<0.001, Two Way ANOVA with Sidak's Post Test R+ *vs.* R-.

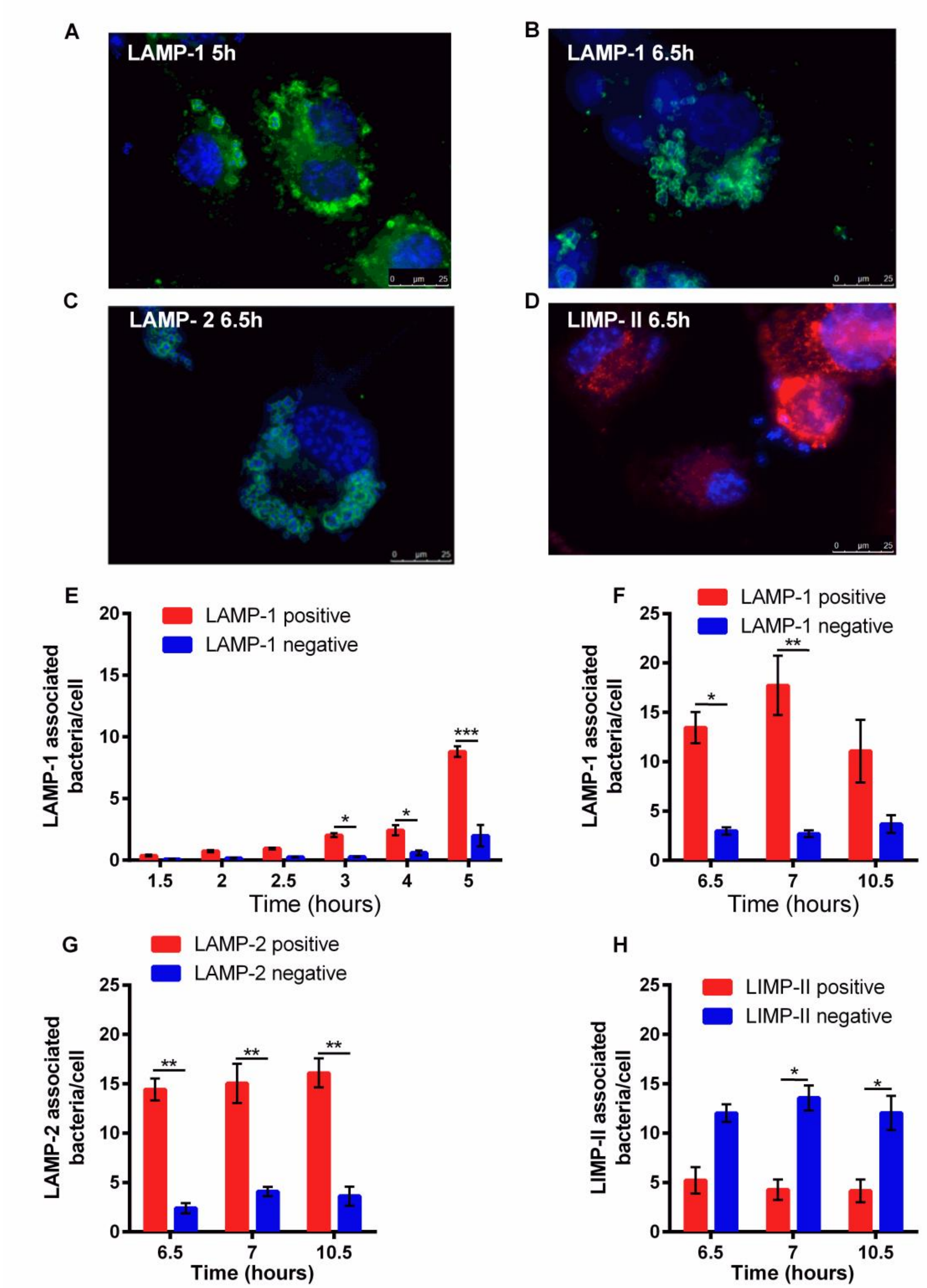

Figure S8: *S. aureus* USA300 JE2 traffic to endosomes which demonstrate incomplete maturation Differentiated THP-1 macrophages were challenged with *S. aureus* USA300 strain JE2, MOI=5. **(A)** Cultures were stained for LAMP-1, LAMP-2 or LIMP-II at the indicated time points. Representative **(A, B)** LAMP-1, **(C)** LAMP-2 and **(D)** LIMP-II staining by confocal microscopy. Number of intracellular bacteria per macrophage co-localizing with **(E, F)** LAMP-1, **(G)** LAMP-2 and **(H)** LIMP-II, at the indicated time points, 3 individual experiments performed in duplicate.*p<0.05, **p<0.01, ***p<0.001, Two Way ANOVA with Sidak's post-test.

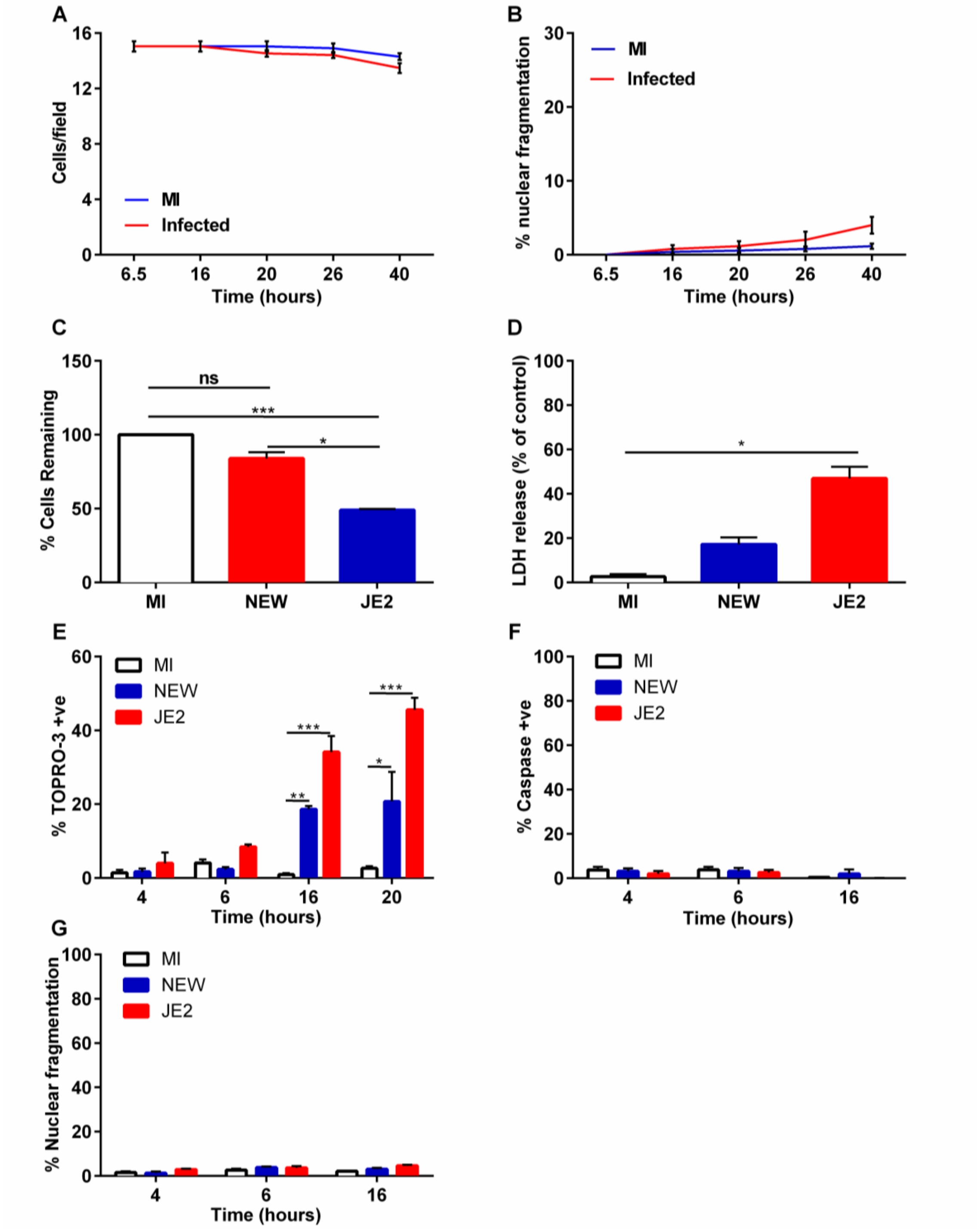

Figure S9: Macrophage apoptosis is not engaged with *S. aureus*. Differentiated THP-1 macrophages were mock-infected (MI) or challenged with *S. aureus* MOI=5 for 6 h. Cultures were treated with lysostaphin and then maintained in low dose lysostaphin for up to 40 h post infection. **(A-B)** MI or challenged with *S. aureus* SH1000 **(A)** Cells per field, **(B)** Percentage of macrophages showing apoptotic nuclei **(C-G)** MI or challenged with *S. aureus* Newman (NEW) or *S. aureus* USA300 JE2 (JE2) for **(C-D)** 20 h or the indicated time points and **(C)** % cells remaining, **(D)** LDH release, **(E)** % TOPRO 3 **(F)** % Caspase 3 positive (+ve) cells and **(G)** % cells with nuclear fragmentation were calculated, 3 individual experiments performed in duplicate. $p<0.05$, $^{**}p<0.01$, $^{***}p<0.001$, Two Way ANOVA with Sidak's post-test.

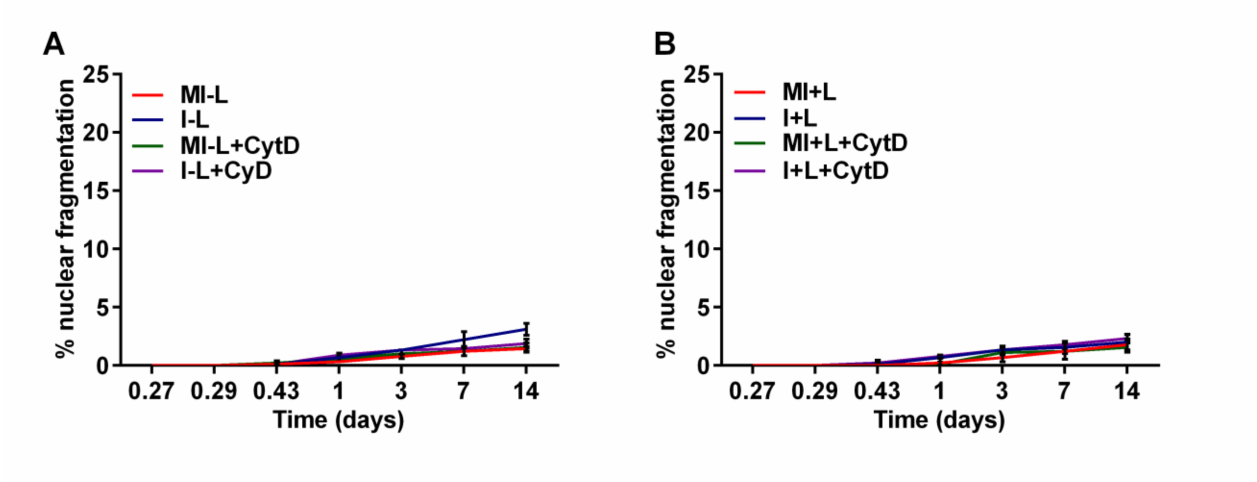

Figure S10: Low macrophage apoptosis is not affected by blocking phagocytosis and killing extracellular bacteria. Differentiated THP-1 macrophages were mock-infected (MI) or challenged with *S. aureus* Newman MOI=0.05 (I) for 6 h. Cultures were treated with lysostaphin (L) and maintained with (+) or without (-) lysostaphin and/or cytochalasin D (CytD) for 1-12 d and stained at the indicated time points. Percentage macrophages with apoptotic nuclei **(A)** without lysostaphin (-L±CytD) and **(B)** with lysostaphin (+L±CytD), 3 individual experiments performed in duplicate.

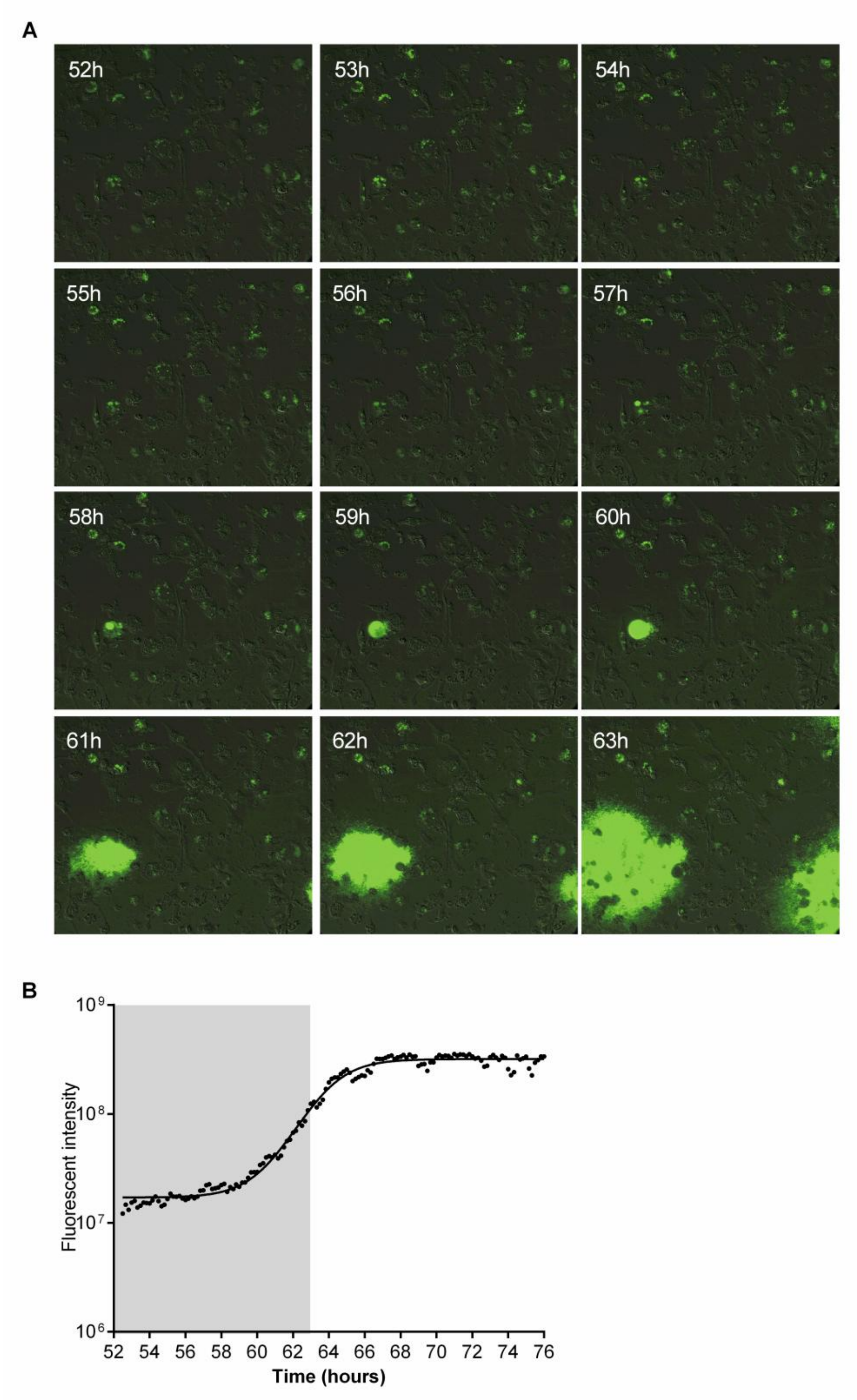

Figure S11: With prolonged culture viable intracellular bacteria replicate and induce macrophage lysis. Differentiated THP-1 macrophages were challenged with *S. aureus* Newman-GFP at an MOI of 5 for 6 h. Cultures were treated without lysostaphin and imaged over 52-72 h. **(A)** Images shown from 52 to 63 h. **(B)** Fluorescent intensity of images, measured by ImageJ, with time period in images in (A) shown in gray.

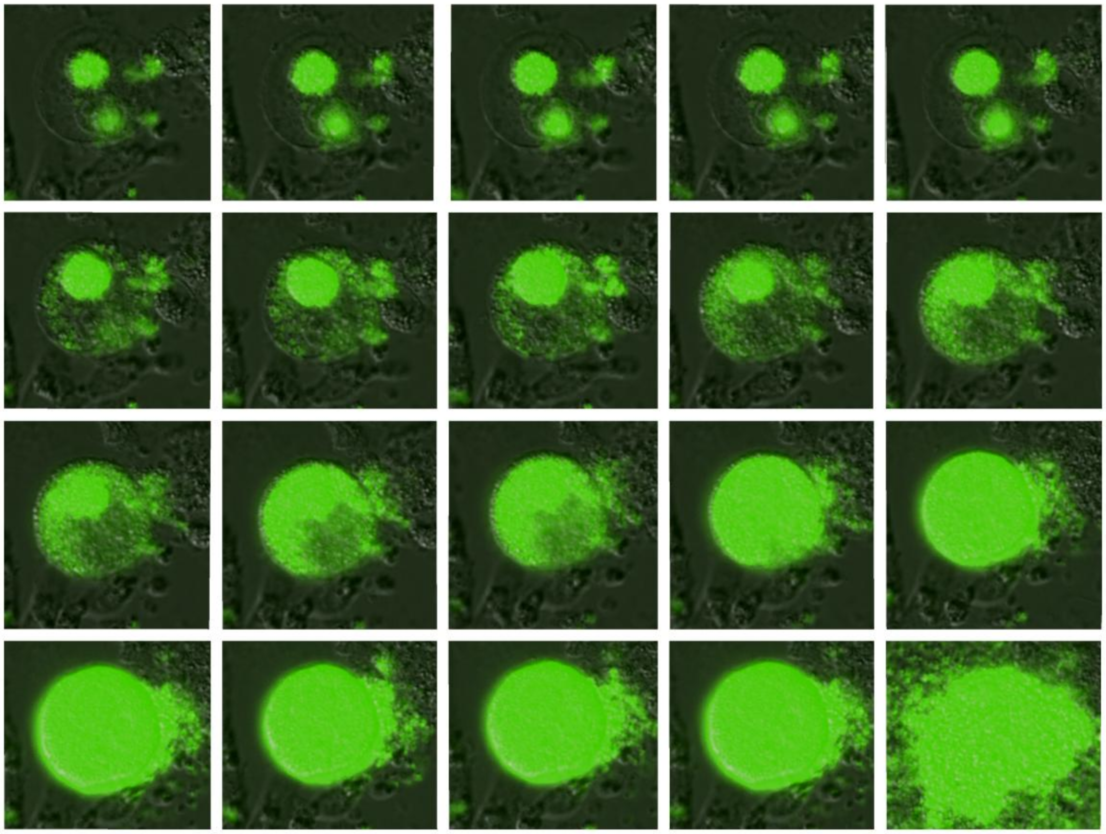

Figure S12: Intracellular replication occurs before lysis. Enlargement of a cell from Figure S11 imaged at 10 min. intervals from 57 h.
